# Beyond the acute phase: a comprehensive literature review of long-term sequelae resulting from infectious diseases

**DOI:** 10.3389/fcimb.2024.1293782

**Published:** 2024-01-31

**Authors:** Juan S. Izquierdo-Condoy, Jorge Vásconez-Gonzáles, Estefanía Morales-Lapo, Andrea Tello-De-la-Torre, Patricio Naranjo-Lara, Ricardo Fernández, Marlon R. Hidalgo, Adriana Escobar, Vanessa Herrera Yépez, Ana María Díaz, Carlos Oliva, Esteban Ortiz-Prado

**Affiliations:** One Health Research Group, Universidad de las Américas, Quito, Ecuador

**Keywords:** post-infectious sequelae, persistent symptoms, chronic complications, global health, sequelae

## Abstract

Infectious diseases have consistently served as pivotal influences on numerous civilizations, inducing morbidity, mortality, and consequently redirecting the course of history. Their impact extends far beyond the acute phase, characterized by the majority of symptom presentations, to a multitude of adverse events and sequelae that follow viral, parasitic, fungal, or bacterial infections. In this context, myriad sequelae related to various infectious diseases have been identified, spanning short to long-term durations. Although these sequelae are known to affect thousands of individuals individually, a comprehensive evaluation of all potential long-term effects of infectious diseases has yet to be undertaken. We present a comprehensive literature review delineating the primary sequelae attributable to major infectious diseases, categorized by systems, symptoms, and duration. This compilation serves as a crucial resource, illuminating the long-term ramifications of infectious diseases for healthcare professionals worldwide. Moreover, this review highlights the substantial burden that these sequelae impose on global health and economies, a facet often overshadowed by the predominant focus on the acute phase. Patients are frequently discharged following the resolution of the acute phase, with minimal long-term follow-up to comprehend and address potential sequelae. This emphasizes the pressing need for sustained vigilance, thorough patient monitoring, strategic health management, and rigorous research to understand and mitigate the lasting economic and health impacts of infectious diseases more fully.

## 1 Introduction

Infectious diseases have left a profound imprint on human history, with the earliest records dating back centuries. From possibly as far back as Roman times, different microorganisms have sparked epidemics and pandemics with devastating consequences (Piret and Boivin, 2021). Some of the pandemics with the highest mortality include Justinian Plague in 541, Black Death in 1348, Spanish Flu from 1918-1919, Human Immunodeficiency virus (HIV) from 1981 to the present, and Severe Acute Respiratory Syndrome Virus Type 2 (SARS-CoV-2) from 2019 to the present (De La Figuera Von Wichmann, 2011; Rius i Gibert, 2019).

The severity or duration of a disease provides a crucial measure of its impact. Acute diseases typically develop swiftly and last a short time, whereas chronic diseases progress slowly, persisting and potentially causing complications or sequelae over long periods. The World Health Organization (WHO) defines complications as a deterioration in disease severity causing systemic organ compromise, while sequelae are conditions that persist after a disease has resolved, potentially affecting the quality of life to varying extents. These outcomes can be influenced by factors such as sex, genetics, nutrition, age, environment, lifestyle, occupation, pre-existing diseases (comorbidities), immunosuppressive therapy, and history of emotional disorders (Tortora, 2017; Wang et al., 2018; World Health Organization, 2018).

Despite efforts to control infectious diseases, numerous outbreaks continue to occur. The emergence of SARS-CoV-2 in Wuhan, China, in 2019 is a prime example. This caught global health systems off guard and had significant global health impacts (Johns Hopkins University, 2020). Through unprecedented efforts, the rapid development of SARS-CoV-2 vaccines has partially helped humanity to overcome the pandemic in less than two years. However, researchers worldwide have identified a range of symptoms and sequelae persisting long after the acute stage of COVID-19 has ended—so-called ‘Long-COVID.’ Currently, ‘Long-COVID’ is regarded as a new pandemic, with projections suggesting that approximately 150 million people worldwide may suffer from the condition by the end of 2022 (Izquierdo-Condoy et al., 2022a; Notarte et al., 2022).

Various microorganisms, including bacteria, viruses, fungi, and parasites, can also cause short-, medium-, and long-term sequelae, affecting the human body’s organs and systems. Examples include Postinfectious glomerulonephritis caused by *Streptococcus pyogenes*, Neurosyphilis by *Treponema pallidum*, Gastric Cancer by *Helicobacter pylori*, Cervical Cancer by *Human Papillomaviru*s (HPV), chronic chagasic cardiomyopathy by *Trypanosoma cruzi*, and Fungal myocarditis by *Candida* spp., among others (Murray, 2013; Bennett et al., 2017).

Given this context, our study seeks to fill a significant gap in the literature by providing a comprehensive review of the sequelae and long-term effects of infectious diseases caused by various pathogens.

## 2 Methods

### 2.1 Research question

We aimed to identify the sequelae most commonly associated with bacterial, viral, fungal, and parasitic infectious diseases.

### 2.2 Study design

In this comprehensive literature review, we incorporated a diverse array of sources, including letters to the editor, editorials, clinical case reports, case series, cohort studies, literature reviews, narrative reviews, systematic reviews, and meta-analyses. Such diverse sources enhanced our overview and understanding of the wide-ranging discussions and data regarding our research question.

### 2.3 Search strategies

The literature search was conducted in both English and Spanish, using databases including PubMed/Medline, SCOPUS, Web of Science, Scielo, Lilacs, and Google Scholar. Additionally, the reference lists of relevant articles were cross-referenced for potential additional resources. Our literature search did not impose any restrictions based on publication date. We employed the following keywords in our search strategy:


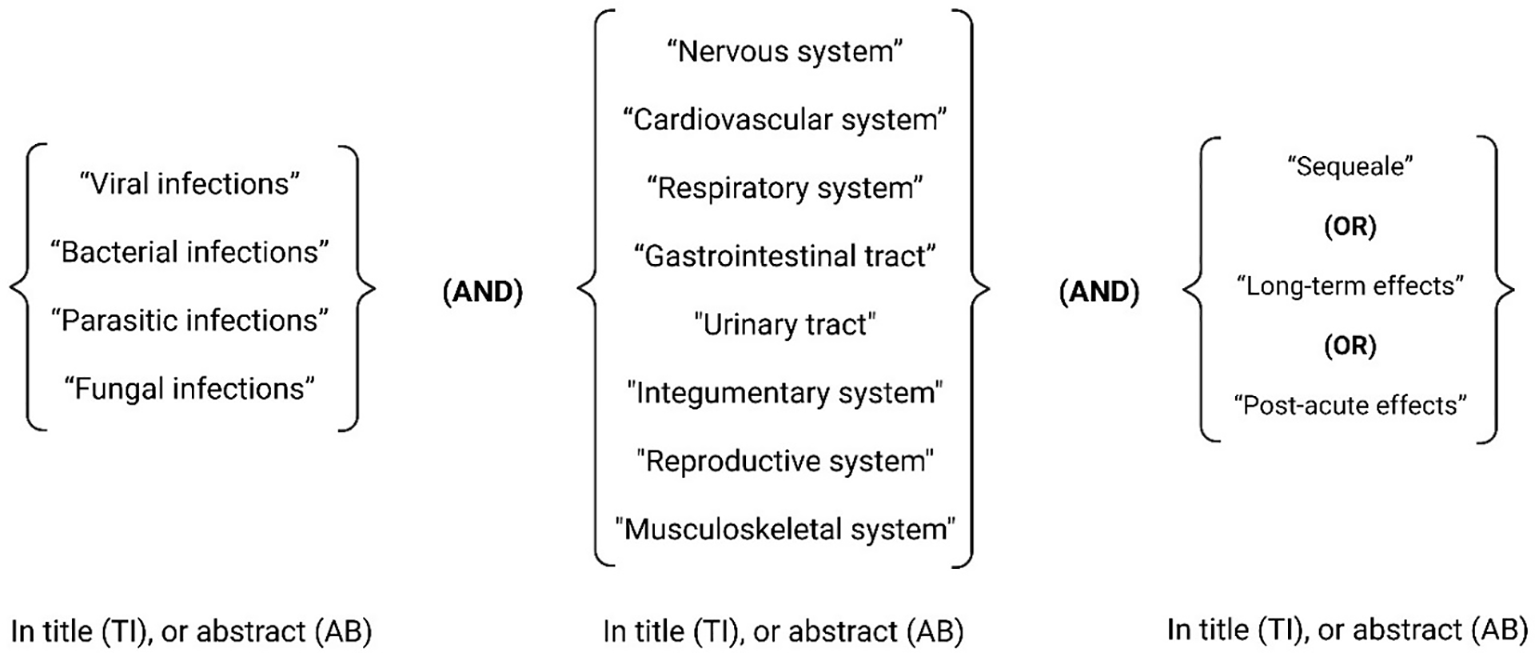


### 2.4 Inclusion criteria

Studies were included if they reported on the sequelae in individuals, affecting any human body system, due to infectious diseases.

### 2.5 Exclusion criteria

Studies were excluded if they were conducted on animal subjects, primarily focused on complications arising from infectious diseases, or reported on exacerbations of pre-existing pathologies triggered by infectious diseases.

### 2.6 Bias assessment

To mitigate bias, the data extraction process was independently conducted by two researchers (JSI-C and JVG) at different times. This independent approach helped ensure a broader perspective and reduced individual bias. Any disagreement or discrepancy in data extraction was resolved including EOP to the discussion, thus, enhancing the reliability of our data extraction.

### 2.7 Data synthesis

In this phase, we meticulously reviewed all studies that met the inclusion criteria to assess their relevance to our research objectives of identifying and classifying sequelae associated with various types of infectious diseases. For an overall assessment of the etiology and severity of the sequelae, four authors (JSI-C, JVG, EM-L, and M-H) independently analyzed each included symptom. In the etiology analysis, we categorized symptoms into four groups based on the causative organism: bacteria, viruses, parasites, and fungi.

We proposed a severity classification adhering to these parameters:

a) *mild sequelae*, which do not interfere with daily activities; b) *moderate sequelae*, which limit daily activities; c) *severe sequelae*, which lead to permanent disability; d) *very severe sequelae*, which can result in death.

## 3 Post infectious sequalae

Infectious disease can cause a variety of sequalae in virtually every organ and system of the human body. Nevertheless, some sequalae can be more deleterious than others, thus, the sequalae affecting the nervous system will be analyzed first, followed by other systems.

### 3.1 Nervous system

#### 3.1.1 Bacteria

The Central Nervous System (CNS) is one of the most protected systems in terms repelling infections, mainly because of the blood-brain barrier, an impermeable shield that act not only blocking microorganisms directly but maintaining sterility despite potential blood contamination or adjacent infection (Dando et al., 2014a). When innate protection fail and the barrier becomes permeable, a variety of microorganism and pathogen might entry to the brain or the spine, affecting surrounding neural tissue leading to potentially lethal damage that not only can lead to acute deterioration, but to short and long term sequelae and death (Dando et al., 2014b).

Bacterial meningitis, the most common CNS infection that often results in long-lasting sequelae. The etiology is diverse and florid depending on the host’s age. Key pathogens such as *Escherichia coli*, Group B *β-hemolytic Streptococcus* (*S. pyogenes*), *Haemophilus influenzae*, *Streptococcus agalactiae*, and *Listeria monocytogenes*, are often linked to severe disease, commonly observed sequelae and death (Olmedo Díaz et al., 1997; Fernández Colomer et al., 2010; Suthar and Sankhyan, 2019).

Meningitis caused by *H. influenzae* is a potently life-threatening disease that is associated with short and long term sequelae, such as hydrocephalus, hemiplegia, paresis, ataxia, and developmental delay (Sproles et al., 1969). Meanwhile meningitis caused by *Streptococcus pneumoniae* may be related to hearing loss (11%), hydrocephalus (11%), focal neurological deficit (3-14%), seizures (7%), cognitive impairment (7%), behavioral disturbances (4%), and visual disturbances, such as strabismus (4%) among children (Lucas et al., 2016; Suthar and Sankhyan, 2019). Although most infections occur among children, when meningitis affects adults, the sequalae such as hearing loss, neurological deficits, and cognitive alterations, especially when caused by germs such as *Neisseria meningitidis* or *S. pneumoniae* can last for long periods of time or even become permanent (Lucas et al., 2016; Suthar and Sankhyan, 2019). Less frequently but not less important, bacteria such as *Mycobacterium tuberculosis* when infecting the brain can cause tuberculous meningitis, a condition that can trigger long-term blindness, deafness, intracranial calcifications, paraplegia or hemiplegia, weakness and decreased coordination of the extremities, epilepsy, hydrocephalus or even diabetes insipidus (Lorber, 1958; Todd and Neville, 1964; Kennedy and Fallon, 1979; Hanna et al., 1988; Palacio Sanguino and Arteaga, 2020).

**Figure 1:**
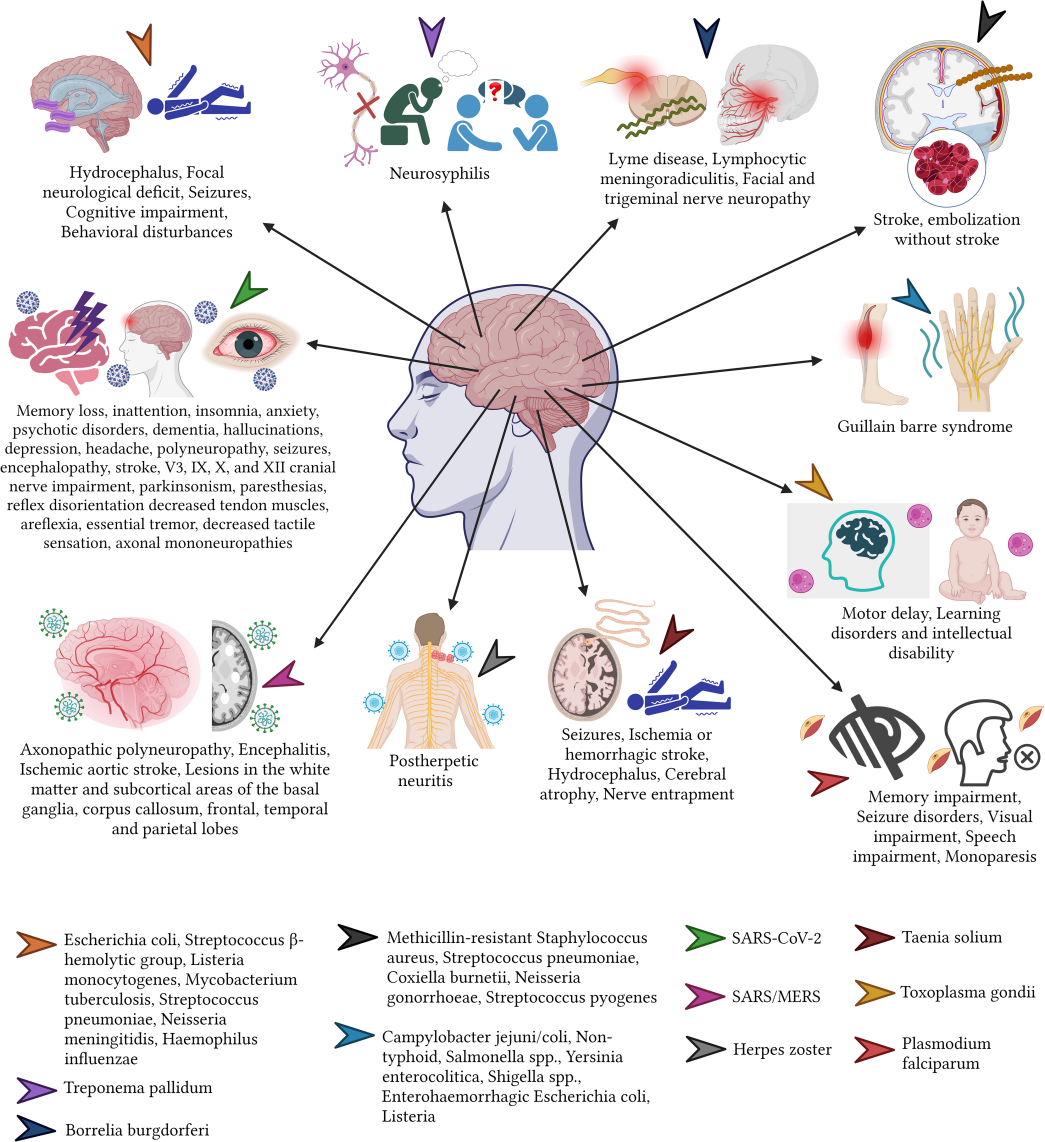
Sequelae of infectious diseases in the nervous system.

Another example of a potentially neurotropic bacteria is *T. pallidum.* This microorganism, when not treated can invade the CNS causing Neurosyphilis (Ghanem, 2010). This late stage of syphilis has been associated with irreversible conditions where “*Tabes dorsalis*” is the most peculiar, damaging the tissue and producing a variety of signs and symptoms (Ropper, 2019; Nassar Tobón et al., 2020; Bhandari et al., 2022) (**Figure 1**).

In the peripheral nervous system, Lyme Disease, caused by *Borrelia burgdorferi* a common disease affecting more than 14% world’s population can lead to a wide range of peripherical nervous sequalae including localized (mononeuropathies) or diffuse polyneuropathies, that typically occur one or two months after the onset of the acute infection (Halperin, 2003; Brizzi and Lyons, 2014; Dong et al., 2022). It is important to point out that some post-infectious radiculopathies, can result in severe neuropathic pain and irreversible atrophy of the affected dermatomes, causing a severe sequela known as Lymphocytic Meningoradiculitis or Bannwarth Syndrome (Halperin, 2016).

A wide variety of microorganism, including bacteria such as *M. Tuberculosis* can affect the cranial nerves (Alvarado Guevara and Castillo Solano, 2006). One of the most common neural affections worldwide is Facial Neuropathy, a condition that often leads to long-term complications such as facial palsy, also known as Bell’s palsy generating physical, psychological, and emotional burden among the affected patients. Less commonly but often more severe are those conditions that did derivate from the involvement of the Trigeminal or the vestibulocochlear nerve, since both can trigger severe chronic pain can (trigeminal neuralgia) or hearing loss (Brizzi and Lyons, 2014; Halperin, 2016).

**Figure 2:**
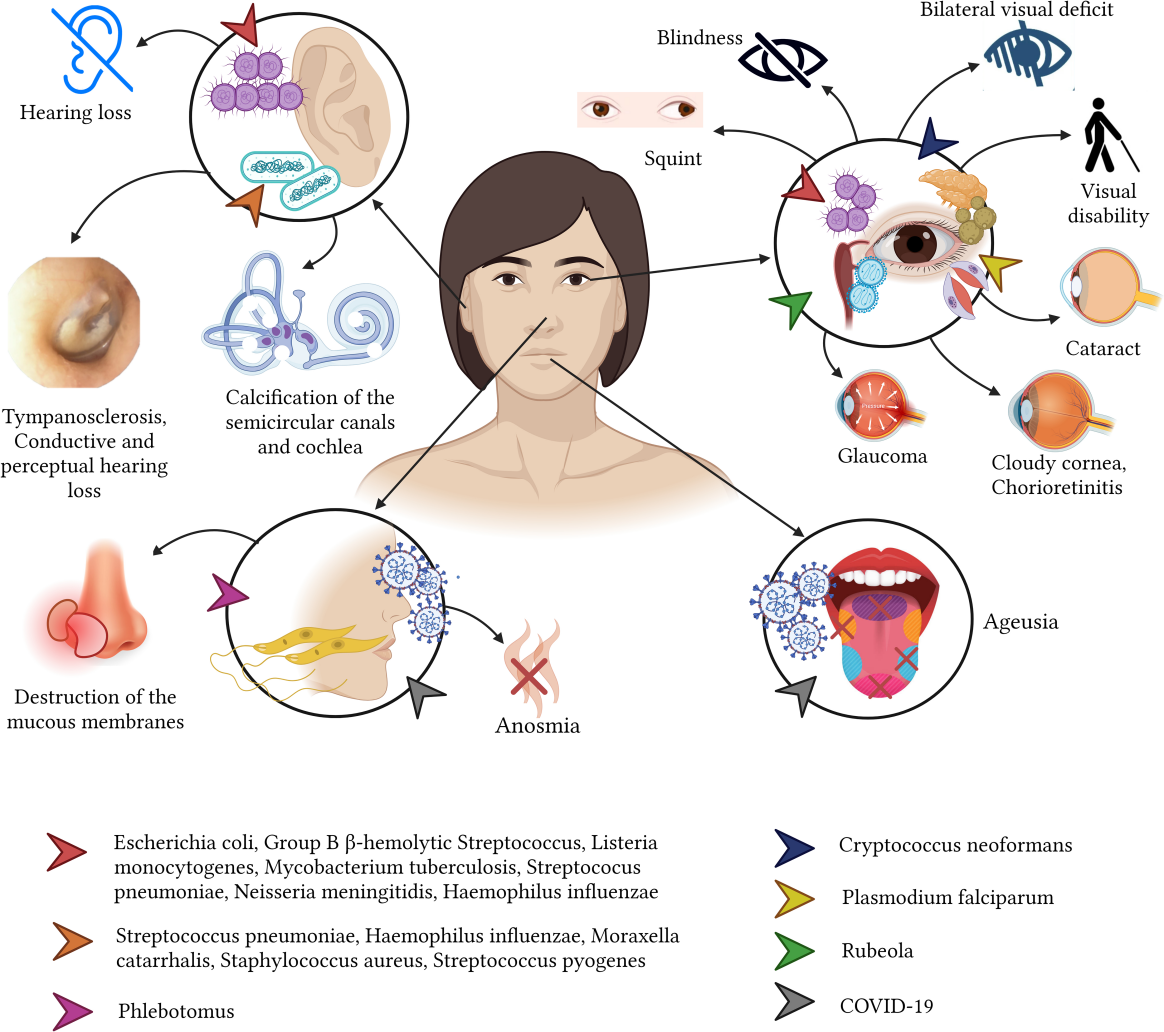
Sequelae of infectious diseases of the eye and ear.

In terms of sense organs such as the eyes, the ears or the nose, the newborns are at the highest risk of severe infections. For instance, *Chlamydia trachomatis* or *Neisseria gonorrhoeae* can cause Neonatorum Ophthalmia from the direct seed of bacteria when the newborn is passing the mother’s vaginal channel (Graue Wiechers, 2014; Arellano Barriga et al., 2020). This condition causes severe complications and therefore serious sequalae among the newborns. For example, when *C. trachomatis* is isolated, the most common sequelae include corneal neovascularization and scarring, leading to loss of corneal transparency and reduced visual acuity (Mårdh, 2002; Mullick et al., 2005). While, ocular infections caused by *N. gonorrhoeae* infections can lead to corneal ulceration and blindness (Turati and Turati, 2000; Mullick et al., 2005; Petour et al., 2016) (**Figure 2**).

When bacteria reach the ear, often caused by a complicated upper respiratory tract infection, the middle ear (more common) and the inner ear can be affected. Bacteria such as the commonly found *S. pneumoniae* and, less commonly the *H. influenzae* or *Moraxella catarrhalis* can cause acute complications including acute otitis media, tympanosclerosis, ossicular fixation and cholesteatomas. When these are not treated promptly, they can cause consequences such as partial or complete sensorineural hearing loss, which are often irreversible (Bluestone, 2000; Casamitjana et al., 2002; Manrique, 2020) (**Figure 2**).

#### 3.1.2 Virus

Virus is elusive microorganism that can affect the nervous system, when a virus is suspected to cause affection of the brain or the meninges, a variety of conditions can be found such as a viral encephalitis or a viral meningitis (Bohmwald et al., 2021). A wide variety of viruses can be considered responsible for these infections such as *Herpes types 6* and *7*, *West Nile*, enteroviruses, *Varicella-Zoster*, *Epstein-Barr*, *cytomegalovirus*, *measles*, *mumps*, *Rubivirus* (rubella virus), *St. Louis*, *eastern equine*, *western equine*, dengue and rabie*s* virus or even *hepatitis B* and *C virus* (Kumar et al., 2018; Soung and Klein, 2018; Said and Kang, 2022). Interestingly enough, the SARS-CoV-2, responsible for the latest pandemic which emerged in 2019 and was declare pandemic in 2020, has been shown to produce a great variety of neurological symptoms that also are linked to the persistence of short, medium and long term sequelae (Izquierdo-Condoy et al., 2022b). After having more than 700 million confirmed COVID-19 cases worldwide, millions of people are dealing with anosmia, dysgeusia, chronic neural pain, persistent headaches, parkinsonisms, disorientation, decreased attention and mental tiredness (brain fog) (Peterson et al., 2021; Mahmood et al., 2022; Mattioli et al., 2022). Similar consequences but to less extended have also been observed in other coronaviruses like SARS-CoV-1 or and (Middle East Respiratory Syndrome) MERS-CoV (Alshebri et al., 2020). For instance, an important percentage of patient with acute MERS-CoV infection can develop white matter and subcortical lesions affecting the basal ganglia, the corpus callosum, and the frontal, temporal, and parietal lobes (Gholami et al., 2021; Peterson et al., 2021; Izquierdo-Condoy et al., 2022a; Mahmood et al., 2022; Mattioli et al., 2022).

Viral infections not only impact physical health but have also been demonstrated to elicit mental sequelae. In the case of SARS-CoV-2, numerous conditions have been reported to affect the psychological and emotional state, including depression, insomnia, anxiety, psychotic disorders, dementia, post-traumatic stress disorder, difficulty concentrating, and hallucinations (Romero-Duarte et al., 2021; Taquet et al., 2021; Damiano et al., 2022; Izquierdo-Condoy et al., 2022a).

Other virus that are currently less likely to progress to more advance stages of the diseases due to the advancement of therapy and diagnosis is the human HIV type 1. This retrovirus when progress to Acquired immunodeficiency syndrome (AIDS) can produce severe neurological affection such as meningoencephalitis that when complicated can produce cognitive dysfunction, epilepsy and other peripherical affections such as Guillain-Barre and other neuropathies that can last for week, months or years (Hogan and Wilkins, 2011; Brew and Garber, 2018; Justiz Vaillant and Gulick, 2022).

In terms of peripherical nerve affection, there is no other virus more commonly found than the Herpes Zoster virus. This can lead to skin eruptions which are accompanied by sharp neurological pain leading to a chronic sequala known as postherpetic (Curran et al., 2018).

Finally, many viruses such as the *cytomegalovirus*, *Zika virus*, and *Rubivirus* can cause congenital conditions affecting the central or peripherical nervous system including the congenital rubella syndrome (CRS) and the microcephaly (PAHO, 2022b). These conditions can lead to numerous nervous sequelae, including cataracts, pigmentary retinopathy, infantile glaucoma, cloudy cornea, chorioretinitis, iris hypoplasia, tear drainage abnormalities, and microphthalmia (Cordier et al., 2012; Bouthry et al., 2014; Lambert et al., 2015).

#### 3.1.3 Parasites

Some parasitic diseases are well known to cause significant neurological complications that can lead to short, medium and long term sequalae (Carpio et al., 2016). One of these examples is the well know neurocysticercosis, caused by *Taenia solium* parasite. When the egg is swallowed and reaches the bloodstream, it is distributed in different tissues, becoming deposited in its larval form and due to the tissue conditions, it can produce a cyst. In the cases that the cyst (or cysts) occupies a portion of the brain, they can lead to the development of seizures and epilepsy, stroke, hydrocephalus, cerebral atrophy, nerve compression, and visual impairment (Del Brutto, 2022). Some recent studies indicate high frequency of depression and cognitive alterations in these patients (Sotelo, 2003; Garcia et al., 2014; Nash and O’Connell, 2020; Bustos et al., 2021; Del Brutto, 2022).

Another relatively common neurotropic parasite especially among susceptible populations is the *Toxoplasma gondii*, a zoonotic virus usually asymptomatic in healthy adults than can lead to serious affections immunocompromised patients or those receiving immunosuppressive therapy (Sanchez and Besteiro, 2021). Latent toxoplasmosis has been correlated with an increased incidence of neuropsychiatric disorders including cryptogenic epilepsy, schizophrenia, headaches and migraine, and attention deficit disorder (Flegr et al., 2014). In children with congenital toxoplasmosis, the infection can lead to psychomotor delay, learning and intellectual disability (Flegr et al., 2014; Basavaraju, 2016; Ayoade and Chandranesan, 2022; Kota and Shabbir, 2022).

Less commonly reported we found that Hydatidosis, caused by *Echinococcus*, is another parasite that can lead to sequelae such as paresthesia, paraplegia, and Brown/Sequard syndrome (Fares et al., 2003; Gessese, 2020). In the same context, schistosomiasis a neglected tropical disease (Vásconez-González et al., 2023c) caused by *Schistosoma mansoni* and *Schistosoma haematobium* (related to spinal cord involvement) could trigger the spinal cord with multiple nodules, or by meningeal granulomas and necrosis of the spinal cord (Walker and Zunt, 2005). On the other hand, *Schistosoma japonicum* infection (related to encephalic involvement) may cause long-term necrosis and rupture of the vascular wall of the leptomeningeal vessels, and consequently cerebral hemorrhages, sometimes resulting in a brain tumor effect causing symptoms of intracranial hypertension and flaccid paraplegia (Carod-Artal, 2010; Ferrari and Moreira, 2011; Carpio et al., 2016; Ramos Romero and Garcia, 2021).

A study on Tanzanian school children found that severe *S. haematobium* infections were linked to cognitive impairment that produced short-term memory loss and information processing speed alteration (Jukes et al., 2002).

Lastly, malaria, one of the most lethal diseases worldwide can lead on cerebral malaria on those who survived. This infestation caused by *Plasmodium falciparum*, can cause acute encephalopathy leading to memory impairment, seizure disorders, visual impairment, speech impairment, and monoparesis (Oluwayemi et al., 2013; Garcia et al., 2014; Carpio et al., 2016).

#### 3.1.4 Fungi

Fungal infections to humans are generally rare, as they are mostly opportunistic infections. However, in some cases, some fungi such as *Cryptococcus neoformans* can cause some infection that may affect the central nervous system and is linked with high morbidity rates. It causes vision loss, mainly attributed to the variant known as “gatti”, which commonly strikes on immunocompetent hosts. The vision loss caused by cryptococcal meningitis can result in a bilateral vision deficit, which often has a permanent effect and can vary in severity (Lizarazo et al., 2000). Patients infected with *C. neoformans* var. *neoformans*, in comparison with those infected with var. *gatti*, have been shown to experience fewer neurological sequelae (Lizarazo et al., 2000).

### 3.2 Cardiovascular system

The cardiovascular system, essential in blood circulation, is exposed to various pathogens leading to serious bacterial, viral, parasitic, and fungal infections with lasting impacts (Tortora, 2017).

#### 3.2.1 Bacteria

There are several bacterial infections that can affect the heart and the circulatory system, predominantly causing bacteremia, nevertheless, within the heart endocarditis, pericarditis or myocarditis are the commonest(Cecchini and González Ayala, 2011). Endocarditis is a clinical syndrome characterized by an infection of the surface of the endocardium with greater involvement of the heart valves, septum, and mural endocardium; mainly caused by methicillin-resistant *Staphylococcus aureus*, followed by *S. pyogenes*, *S. pneumoniae*, *Coxiella burnetii* (cause of Q fever), and *N. gonorrhoeae*. Endocarditis, primarily affecting young (15 to 34 years) intravenous drug users and adults (58 to 77 years) with underlying conditions, can lead to stroke, heart failure, chronic fatigue, intracardiac abscess, and conduction abnormalities, most of these being reversible with adequate treatment (Cecchini and González Ayala, 2011; Baddour et al., 2015; Eldin et al., 2017; Vincent and Otto, 2018; Wang et al., 2018).

Not all the bacteria lead to endocarditis, some of them such as the *M. tuberculosis* can lead to pericarditis, especially in developing countries (López-López et al., 2021). Other bacteria such as *S. pneumoniae*, *H. influenzae* (especially in children), *Mycoplasma pneumoniae*, *C. burnetii*, *B. burgdorferi* can also cause pericarditis and led to unprecedent sequalae (Cecchini and González Ayala, 2011). In general terms, patients that recover from pericarditis might increase their risk of developing recurrent infections (18.3%), cardiac tamponade (3.1%), constrictive pericarditis (1.5%) and increase the risk of ischemic heart diseases (Cecchini and González Ayala, 2011; Imazio et al., 2015).

**Figure 3:**
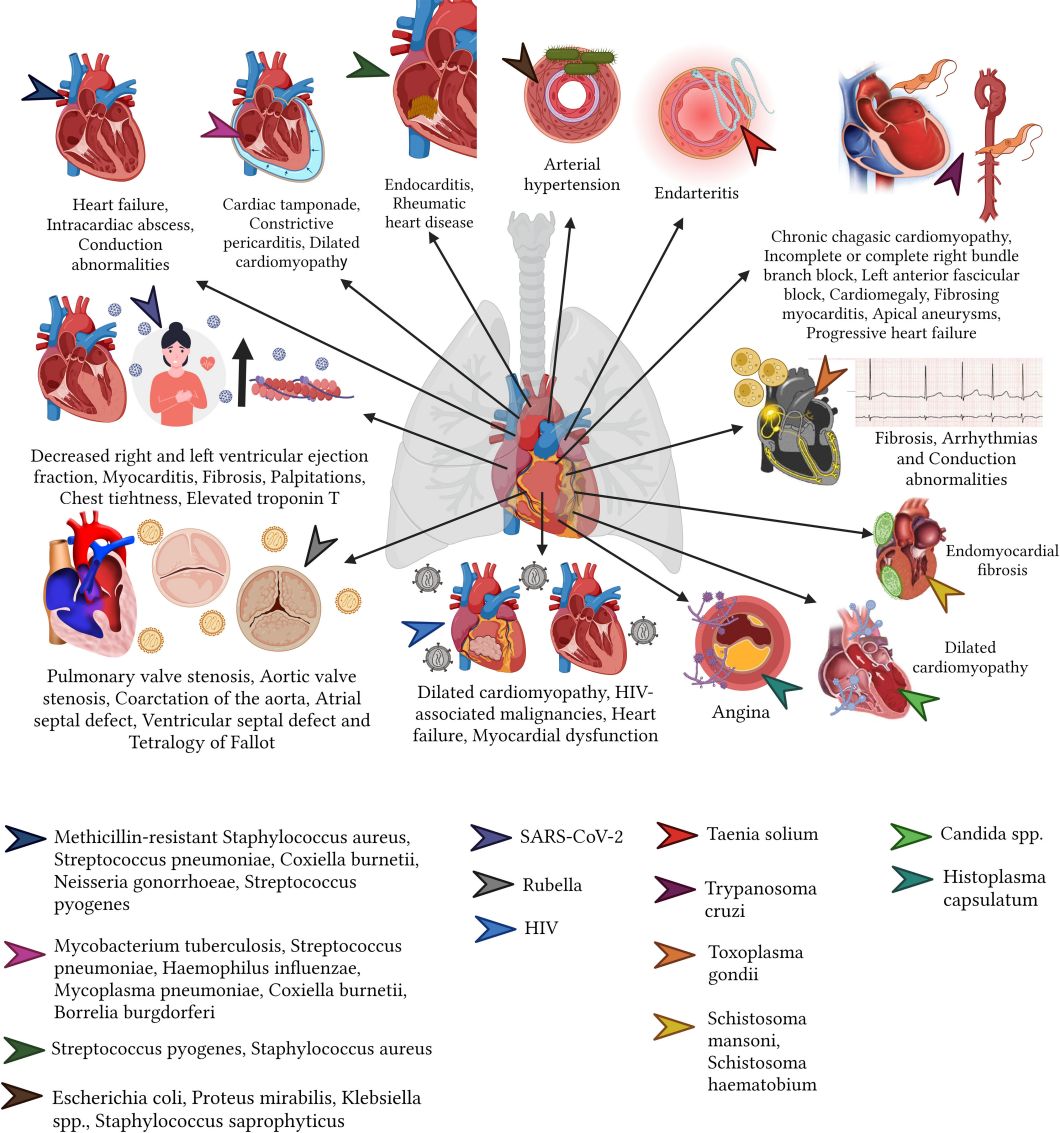
Sequelae of infectious diseases in the cardiovascular system.

Myocarditis, although rare (incidence rate 0.2% to 1.5%), can lead to dilated cardiomyopathy, caused by *S. aureus*, *S. pyogenes*, *S. pneumoniae*, *N. meningitidis*, *Salmonella* spp., *H. influenzae*, *Corynebacterium diphtheriae, M. pneumoniae*, *C. Burnetii*, *T. pallidum, Borrelia* (Lyme disease), SARS-CoV-2 and *Leptospira* (Cecchini and González Ayala, 2011; Eldin et al., 2017; Albakri, 2019a; Mac et al., 2020). Although rare, this disease gained public importance since vaccination was blamed for the appearance of post-covid myocarditis, however the risk of developing myocarditis post-COVID is several times higher among unvaccinated subjects (Patone et al., 2022).

acute rheumatic fever caused by *S. pyogenes* may resolve in the short term; however, valvular damage may persist, and rheumatic heart disease may occur. Although rheumatic heart disease can occur in children, the sequelae typically peak in adults between ages 25 and 45, primarily in women (Carapetis et al., 2016) (**Figure 3**).

#### 3.2.2 Virus

In terms of virus, the heart can be targeted by various viral diseases and cardiotropic viruses. For instance, the *Rubivirus* can cause congenital Rubella Syndrome can result in a variety of cardiovascular sequelae including pulmonary artery hypoplasia, pulmonary valve stenosis, aortic valve stenosis, coarctation of the aorta, atrial septal communication defect, ventricular septal defect, and tetralogy of Fallot (Shukla and Maraqa, 2022). Another example is *Varicella Zoster.* This Virus can lead in adults to ischemic or hemorrhagic cerebrovascular accidents due to arterial vasculopathy and cavernous sinus thrombosis (8.1 to 35.6%. of all thrombosis of the cerebral cavernous sinus) (Alamlih et al., 2021). Less commonly but no less important, *Hepatitis B virus* (HBV) infection can lead to polyarteritis nodosa (20 to 30%) (Faustini et al., 2010).

On top of those viruses, *Arboviruse*, a type pf virus that are commonly associated with vectorial diseases such as Dengue or Chikungunya, can also led to cardiac affections and sequelae, including arrhythmias, myocarditis, pericarditis, pericardial effusion, myocardial injury and heart failure (Bäck and Lundkvist, 2013; Alvarez et al., 2017) (**Figure 3**).

More recently, the appearance of SARS-CoV-2 virus have been greatly associated with significant cardiac malfunctional including decreased ejection fraction in both the left and right ventricle, focal myocardial fibrosis, as well as persistent symptoms such as tachycardia, chest pain, and chest tightness, that although most of them might be reversible, we are still learning and awaiting to see longer term effects (Huang et al., 2020; Puntmann et al., 2020).

#### 3.2.3 Parasites

In relation to parasites, the most common of these organisms capable of affecting the heart is *T. cruzi* (Vásconez-González et al., 2023b). This parasite is associated with chronic chagasic cardiomyopathy that generates hypertrophy of the heart (cardiomegaly), incomplete or complete right bundle branch block, left anterior fascicular block, cardiomegaly, fibrosing myocarditis, apical aneurysms, and progressive heart failure (Marin-Neto et al., 2007; Rassi et al., 2007; Pinto et al., 2008; Meymandi et al., 2018; Martín-Escolano et al., 2022). Other parasites, including Toxoplasma, can cause constrictive pericarditis, arrhythmias, and congestive heart failure. Cysticercosis can also lead to arrhythmias and neural conduction abnormalities, while schistosomiasis can cause left ventricular hypertrophy (Júnior et al., 2002; Hidron et al., 2010); finally, Filariasis and Schistosomiasis induce chronic eosinophilia leading to endomyocardial fibrosis in some rare cases (Martín-Escolano et al., 2022) (**Figure 3**).

#### 3.2.4 Fungi

Immunocompromised patients suffering from invasive candidiasis caused by *Candida* spp., may develop fungal myocarditis, that in a long-term can originate dilated cardiomyopathy as a sequela (Albakri, 2019b; Cleveland Clinic medical professional, 2022).

Acute pulmonary infections caused by *Histoplasma capsulatum* can lead to pericarditis, with symptoms such as fever and angina that may persist for weeks or even months. However, these conditions typically have a good long-term prognosis (Kurowski and Ostapchuk, 2002). However, *C. neoformans* can cause a lethal cardiac affectation (Albakri, 2019b) (**Figure 3**).

### 3.3 Respiratory system

The respiratory system, which includes the pulmonary parenchyma, bronchi, and bronchioles, can manifest lasting sequelae due infectious diseases, especially to bacteria, when they are not addressed promptly (Barker et al., 1991; Edmond et al., 2012).

#### 3.3.1 Bacteria

The risk for permanent sequelae in adulthood is heightened with early onset (during childhood) of bacterial pulmonary infections (Barker et al., 1991; Edmond et al., 2012; Grimwood and Chang, 2015). Bacterial pneumonia in children, resulting from pathogens like *H. influenzae*, *S. pneumoniae*, and *M. catarrhalis*, often leads to bronchiectasis, which is the most prevalent sequel (Pizzutto et al., 2017). Furthermore, bacterial pneumonia caused by *S. pneumoniae* and *H. influenzae* in children from developing nations can result in restrictive pulmonary disease, marked by decreased lung compliance (Edmond et al., 2012; Martinez-Pitre et al., 2022). Additionally, children with a history of bacterial pneumonia due to *S. pneumoniae* have an increased risk of developing asthma (Grimwood and Chang, 2015; Rhedin et al., 2022).

In adults, bronchiectasis and pneumatoceles are most frequently caused by pathogens such as *S. aureus* and Pseudomonas aeruginosa (Daltro et al., 2011; Pizzutto et al., 2017). *M. pneumoniae* infection, often referred to as community-acquired pneumonia, can lead to bronchiolitis obliterans, characterized by inflammation and fibrosis of bronchioles, culminating in respiratory distress (Huang et al., 2017; Zheng et al., 2022).

**Figure 4:**
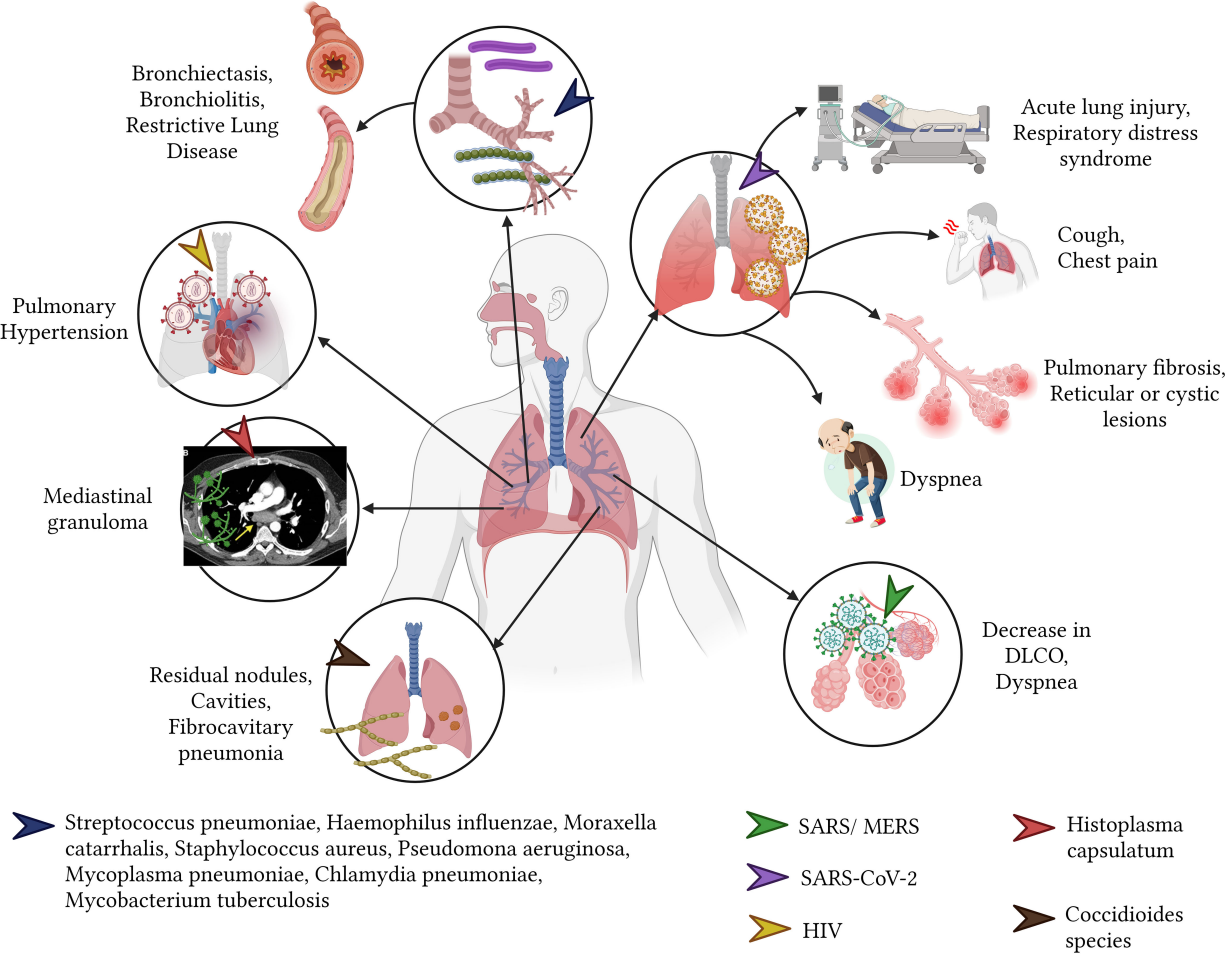
Sequelae of infectious diseases in the respiratory system.

Despite advancements in medical treatments, *M. tuberculosis* continues to pose significant global health challenges with high morbidity and mortality rates, despite advancements in medical treatments, *M. tuberculosis* continues to pose significant global health challenges with high morbidity and mortality rates (World Health Organization, 2021). Sequelae from this infection encompass traction bronchiectasis, intrapulmonary cavern formation leading to sustained loss of lung parenchyma, scarring atelectasis, pleural thickening, mediastinal fibrosis, Rasmussen’s aneurysms affecting the pulmonary vasculature, and broncholiths (Kim et al., 2001; Romero Marín et al., 2016; Aggarwal et al., 2021) (**Figure 4**).

#### 3.3.2 Virus

Members of the coronavirus family, such as SARS-CoV-2, SARS, and MERS, have the potential to impair lung parenchyma, resulting in diminished forced expiratory volume (FEV1) and forced vital capacity (FVC) (Zhao et al., 2020; Huang et al., 2021; Xiong et al., 2021). The aftereffects of these infections include fibrosis, reticular or multifocal cystic lesions, and remaining linear opacities (Pan et al., 2022). Persistent symptoms in the general population, as well as in vulnerable groups like pregnant women, comprise fatigue (the most common symptom at 10.6%), runny nose, dyspnea, cough, sneezing, rhinitis, and chest pain (Carvalho-Schneider et al., 2021; Halpin et al., 2021; Vásconez-González et al., 2023a). SARS and MERS viruses can induce sequelae in the pulmonary parenchyma akin to those caused by SARS-CoV-2, typified by a decrease in diffusion test for carbon monoxide (DLCO) and accompanied by symptoms like fatigue, reduced exercise capacity, and dyspnea (O’Sullivan, 2021) (**Figure 4**).

#### 3.3.3 Fungi

Individuals with pulmonary histoplasmosis by *H. capsulatum* present with symptoms reminiscent of chronic obstructive pulmonary disease due to the involvement of the parenchyma during acute infection (Adenis et al., 2014). Pulmonary fungal infections, predominantly in patients with compromised immune systems, can often have fatal consequences (Adenis et al., 2014). Conversely, acute pulmonary histoplasmosis can induce inflammatory changes in the mediastinal lymph nodes, manifesting as mediastinal granuloma. This condition can lead to symptoms of vascular and airway compression such as cough, dyspnea, angina, and hemoptysis (Kurowski and Ostapchuk, 2002; Sayeed et al., 2022) (**Figure 4**).

Following primary pulmonary infection, Coccidioides species, which include *Coccidioides immitis* and *Coccidioides posadasii*, may cause residual pulmonary nodules that persist for months or years after acute infection symptoms have subsided (Galgiani et al., 2016). Additionally, these infections can lead to the formation of coccidioidal cavities which, when not completely resolved through specific antifungal treatment, may necessitate surgical intervention for complete closure (Galgiani et al., 2016; Panicker et al., 2021). Over the long term, chronic fibrocavitary pneumonia can develop, characterized by pulmonary infiltrates that cause systemic symptoms, including night sweats and weight loss (Galgiani et al., 2016).

### 3.4 Gastrointestinal tract

The gastrointestinal tract is a complex system crucial for the digestion of food and absorption of essential nutrients. However, it is susceptible to various infections and diseases, particularly due to bacteria and parasites, leading to an array of potential sequelae.

#### 3.4.1 Bacteria

Bacterial infections of the gastrointestinal tract can range from minor irritations to severe diseases. *H. pylori* infection is currently considered the leading cause of chronic inflammatory processes and stomach malignancy-related sequelae. *H. pylori* can be found in up to 80% of middle-aged adults in developing countries, though most remain asymptomatic. However, the rest may exhibit acute conditions like gastritis, functional dyspepsia, and gastroesophageal reflux, with subsequent sequelae including chronic inflammation, peptic ulcers (10%), gastric cancer (1-3%), and mucosa-associated lymphoid tissue lymphoma (MALT) (0.1%) (Wang et al., 2014).

Bacteria such as *Campylobacter jejuni/coli*, non-typhoidal *Salmonella* spp., *Yersinia enterocolitica*, *Shigella* spp., Enterohemorrhagic *E. coli*, and *Listeria* can lead to foodborne illnesses. These can manifest in sequelae such as irritable bowel syndrome, malabsorption syndrome, Crohn’s disease, dyspepsia, reactive arthritis, ulcerative colitis, pancreatitis, liver failure, and Guillain-Barré syndrome (Esan et al., 2017). Most of these sequelae become evident or are identified after a year and are predominantly irreversible (Pogreba-Brown et al., 2020).

**Figure 5:**
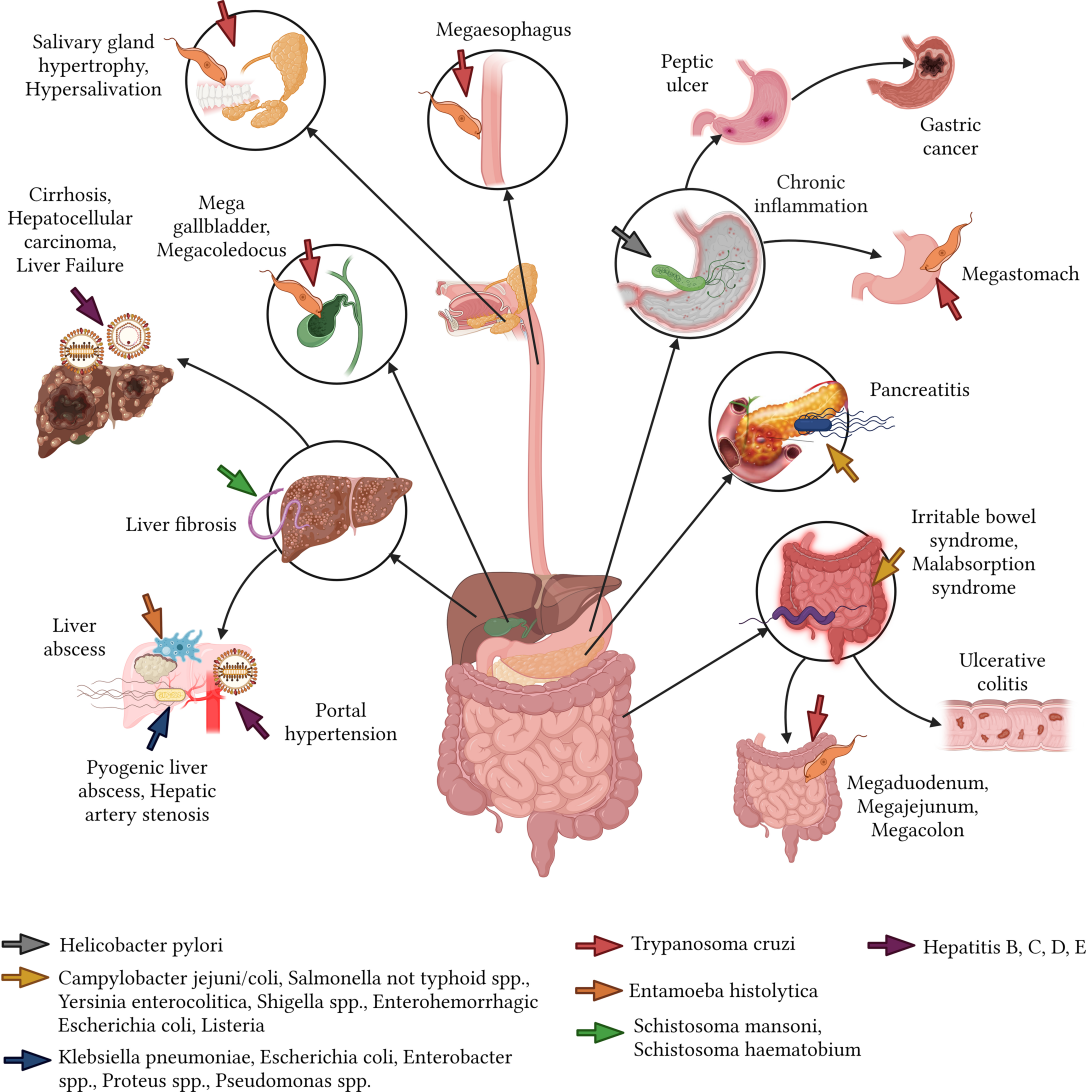
Sequelae of infectious diseases in the gastrointestinal tract.

The pyogenic liver abscess, a pus-filled mass in the liver can arise from a variety of bacterial sources. For instance, infections from *Klebsiella pneumoniae* (54%), *E. coli* (29%), *Enterobacter* spp. (9%), *Proteus* spp. (6%) and *Pseudomonas* spp. (5%), may lead to sequelae, including hepatic artery stenosis, rhabdomyolysis, chronic renal failure, endophthalmitis, and meningitis, especially in individuals aged between 65 and 84 years (Roediger and Lisker-Melman, 2020) (**Figure 5**).

#### 3.4.2 Parasites

Parasitic infections in the gastrointestinal tract can have debilitating effects, often leading to chronic conditions that affect a person’s quality of life. Chagas disease, induced by the protozoan *T. cruzi*, can result in medical complications for near to 30% of affected individuals years after the initial infection (Matsuda et al., 2009). Notable sequelae include megaesophagus, salivary gland hypertrophy, sialorrhea, and megastomach with abnormal gastric emptying (Boyce and Bakheet, 2005; Matsuda et al., 2009). Additional sequelae encompass small intestine dilation such as megaduodenum and megajejunum (Troncon et al., 2000), and megacolon, which leads to chronic constipation (Bern et al., 2007). Other observed changes include mega gallbladder and megacoledochus (Matsuda et al., 2009). In the context of chronic schistosomiasis, hepatosplenic involvement can escalate to severe liver fibrosis (Martín-Escolano et al., 2022). Digestive sequelae are predominantly observed in South American nations like Brazil, Argentina, Bolivia, Chile, and Paraguay, while they are considerably rarer in northern Latin American regions such as Mexico and Central America (Stanaway and Roth, 2015; Meymandi et al., 2018) (**Figure 5**).

### 3.5 Integumentary system

The integumentary system, comprising the skin and its appendages, acts as a protective barrier against external threats. It’s essential to understand how various pathogens affect this system and their potential aftermath.

#### 3.5.1 Bacteria

**Figure 6:**
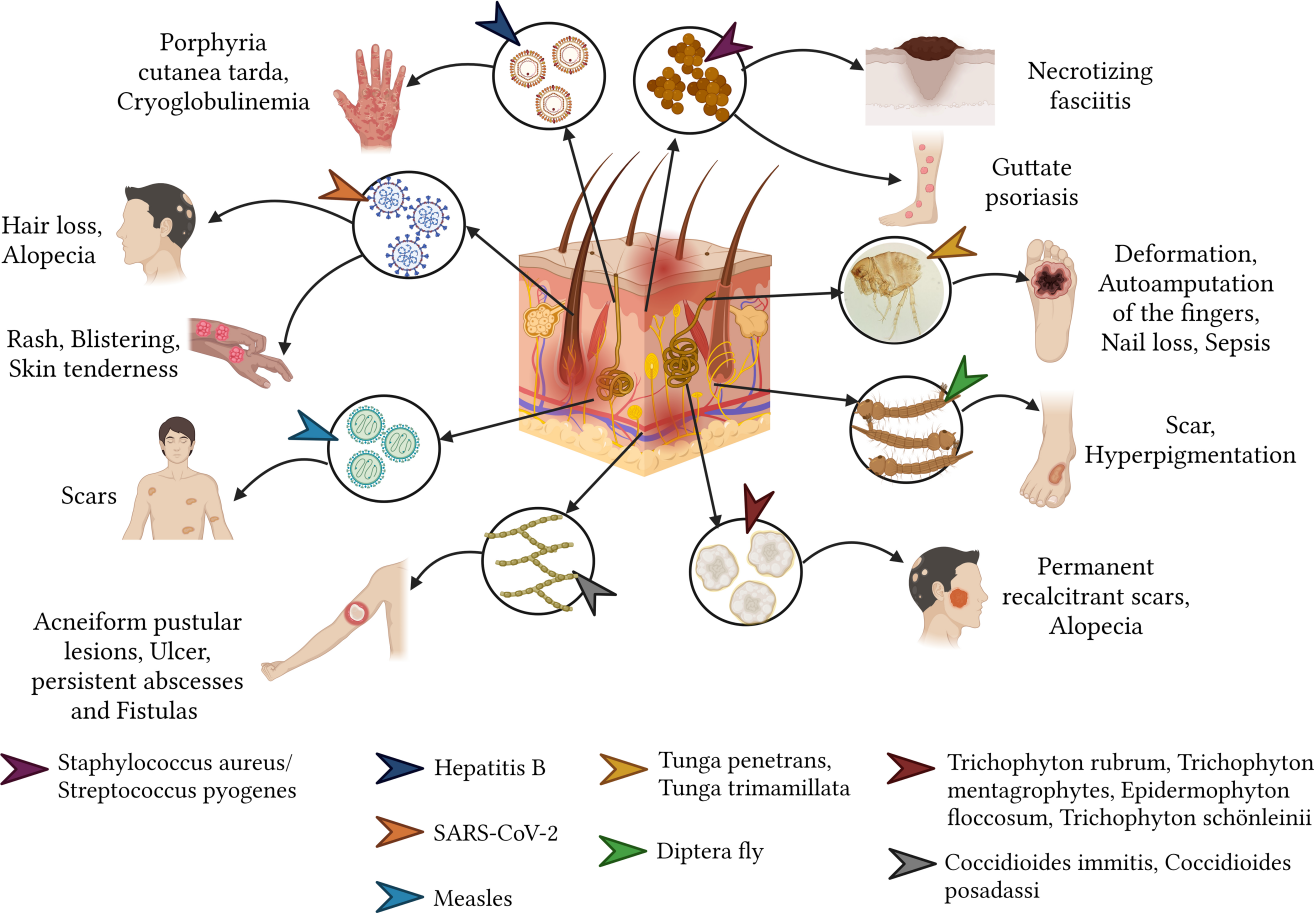
Sequelae of infectious diseases in the integumentary system.

The skin, recognized as the body’s largest organ, provides a niche for countless bacterial species. Disruptions in the skin’s integrity can give rise to acute infections, with the outcomes hinging on bacterial virulence factors, the host’s immunity, and extrinsic conditions (Tognetti et al., 2012). Predominant pathogens like *S. aureus* and *S. pyogenes* often trigger skin infections, with possible ensuing complications (Berger, 1993; Strange et al., 1996; Chiller et al., 2001; Murray, 2013). For instance, infections from *S. pyogenes* in younger populations can manifest as Guttate Psoriasis Conversely, *S. aureus* in similar demographics might result in afflictions like impetigo and erysipelas (Chiller et al., 2001; Bolognia et al., 2015; Cemeli Cano and Beltrán García, 2019) (**Figure 6**).

#### 3.5.2 Virus

In addition to bacterial agents, the skin can also reflect the presence of viral invaders. Post-infection sequelae from viruses, notably SARS-CoV-2, encompass symptoms like hair loss (effluvium telogen) between 1 to 3 months after infection (affecting approximately 42.3% of women and 6.2% of men), rash, blisters, skin tenderness, and alopecia areata (Aksoy et al., 2021; Anaya et al., 2021). Moreover, the aftermath of a measles virus infection can include enduring scars (Patterson, 2020) (**Figure 6**).

#### 3.5.3 Parasites

Parasitic invasions of the skin, such as Leishmaniasis, transmitted via *Phlebotomus* sandfly bites, can lead to permanent scarring and reduced quality of life due to cutaneous leishmaniasis, causing psychological suffering and stigmatization (Sundar and Chakravarty, 2015; Bilgic-Temel et al., 2019). Meanwhile mucosal leishmaniasis can lead to partial or complete destruction of the nose and mouth mucous membranes, causing severe disability (PAHO, 2022a). Other parasites like *Tunga penetrans* and *Tunga trimamillata* (sand fleas), can penetrate the skin causing tungiasis, leading sequelae like difficulty walking, deformations, loss of toenails, and self-amputation of toes (Ariza et al., 2007; Feldmeier et al., 2014; Carretero-Anibarro and Peñacoba-Masa, 2022; Ebrahim et al., 2022) (**Figure 6**). Another notable condition is Myiasis, a skin infestation stemming from Diptera fly larvae, which can result in skin discolorations or scars (Francesconi and Lupi, 2012; Calvopina et al., 2020).

#### 3.5.4 Fungi

Despite being less common, fungal pathogens can wreak havoc on the skin. Dermatophytosis of the skin affecting the hands and feet, caused by *Trichophyton rubrum* (lineage A-4), *Trichophyton mentagrophytes* (anthrophilic and zoophilic species lineage A-1), and *Epidermophyton floccosum* (paraphyletic species lineage B), can lead to permanent recalcitrant scars (Veien et al., 1994; de Hoog et al., 2017). The “Favic ringworm”, caused by *Trichophyton schönleinii* (lineage A-2), can cause permanent scarring alopecia if untreated (Bennett et al., 2017; de Hoog et al., 2017). In addition, Lymphocutaneous sporotrichosis can leave indelible injuries from the original infection (Mahajan, 2014). Finally, the mycetoma caused by fungus organisms, can deform the affected area and leave painful scar injuries, permanent nodes and fistulae (Relhan et al., 2017).

Cutaneous coccidioidomycosis, mainly due to *C. immitis* and *C. posadasii*, has been associated with a range of chronic skin manifestations, including gum-like sequelae, acneiform pustular lesions, and ulcerated, warty plaques, as well as persistent abscesses and fistulas. These varied lesions can manifest in any cutaneous region, highlighting the infection’s potential for widespread dissemination (Welsh et al., 2012).

Furthermore, cutaneous zygomycosis, resulting from infection by fungi of the *Zygomycetes* class, particularly members of the Mucorales order, is characterized by their propensity to colonize perivascular and intravascular spaces, subsequently invading blood vessels. This invasion can lead to ischemia and infarction, with the grave potential outcome of tissue necrosis, most notably in areas compromised by trauma (Arnáiz-García et al., 2009).

### 3.6 Musculoskeletal system

#### 3.6.1 Bacteria

**Figure 7:**
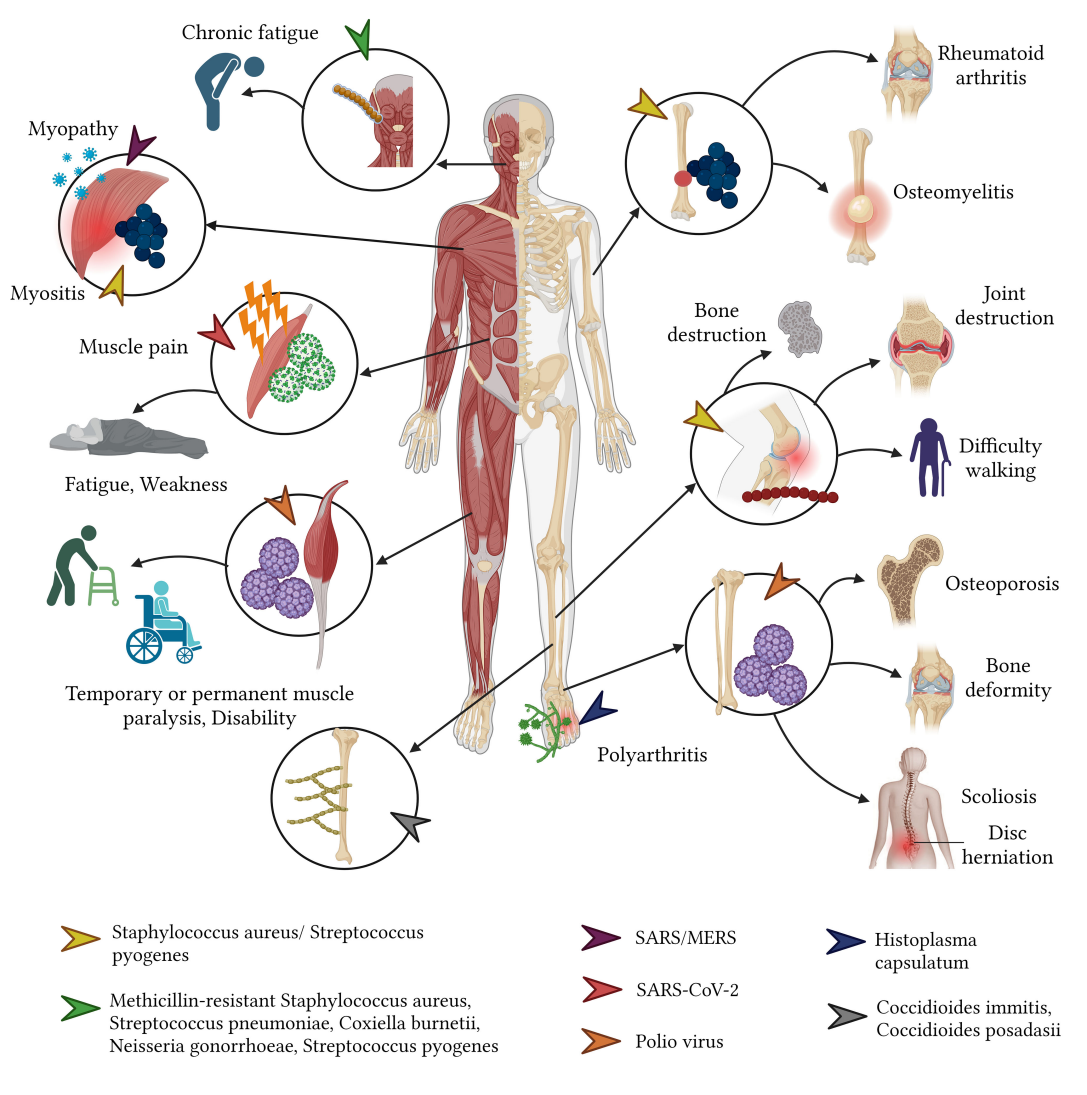
Sequelae of infectious diseases in the musculoskeletal system.

The bacterial invasion of the musculoskeletal system can occur through multiple routes: bloodstream dissemination, direct extension from neighboring infected tissues, direct inoculation, or post-surgical complications. While children predominantly suffer from acute hematogenous osteomyelitis and septic arthritis, adults tend to experience conditions like septic arthritis, periprosthetic joint infections, post-osteosynthesis infections, osteomyelitis, and spondylodiscitis. Predominant bacterial culprits include *S. aureus –* often the methicillin-resistant (MRSA)—followed by *S. pyogenes*, *S. pneumoniae*, and *Enterobacter* spp. Such infections can lead to dire long-term outcomes, such as severe joint and bone destruction. In children, this can culminate in joint necrosis and even amputation, while adults may encounter joint degeneration, osteonecrosis, and disability. Notably, deep vein thrombosis—potentially progressing to pulmonary embolism—is another concerning sequela, often observed in children with specific conditions such as acute hematogenous osteomyelitis and septic arthritis (Vander Have et al., 2009; Godley, 2015; Ochsner et al., 2016; Balato et al., 2021; Saeed et al., 2021) (**Figure 7**).

#### 3.6.2 Virus

Viruses pose their distinct challenges to the musculoskeletal system. Of these, the *poliovirus*, especially serotypes 1, 2, and 3, is notorious for eliciting a slew of musculoskeletal sequelae. Survivors of poliomyelitis often confront a myriad of physical ailments ranging from fixed flexion deformities and joint instability to osteoporosis and osteoarthritis (Howard, 2005; Wolbert and Higginbotham, 2022). Prolonged sequelae such as scoliosis secondary to poliomyelitis might span over four decades, presenting with symptoms like neck pain, spinal discomfort, and even quadriplegia (Winter and Lonstein, 2009). Additionally, the *rubella virus* is known to induce arthralgia and arthritis in a significant proportion (60% - 70%) of adolescent girls and adult women, with other complications such as carpal tunnel syndrome and tenosynovitis being rarer but still consequential (Banatvala and Brown, 2004; Dontigny et al., 2018; Kondamudi and Waymack, 2022) (**Figure 7**).

#### 3.6.3 Fungi

Fungi, while not the most common culprits of musculoskeletal infections, can induce severe complications when they do invade. Osteoarticular infections are frequently caused by fungal species such as *Scedosporium apiospermum*, *Aspergillus*, and *Candida*, and may escalate to conditions necessitating limb amputation in extreme cases (Taj-Aldeen et al., 2015). Acute pulmonary histoplasmosis is another fungal affliction that can give rise to post-acute, widespread inflammatory reactions, leading to symmetrical polyarthritis, which is amenable to anti-inflammatory treatment (Kurowski and Ostapchuk, 2002; Sayeed et al., 2022) (**Figure 7**). Typically, fungal infections target immunocompromised individuals and can disseminate to various body parts including the skin, lungs, bones, and joints. This widespread invasion often portends a grim prognosis, with the exception being those who receive a timely diagnosis and treatment or those not suffering from a disseminated infection.

In cases of disseminated coccidioidomycosis caused by *C. immitis* and C. *posadasii*, the resulting sequelae arise from infections of tendons, joints, and bones, predominantly affecting the thoracic and lumbar spine, tibia, skull, metatarsals, metacarpals, femur, and ribs. Sequelae such as lytic bone lesions and occasionally cysts can be detected via computed tomography (Welsh et al., 2012).

Furthermore, when unrecognized or inadequately treated, candidiasis caused by *Candida* spp. has the potential to develop into osteomyelitis of the spine, with or without discitis. This condition can lead to various sequelae, including chronic local back pain and nerve compression syndrome (McCarty et al., 2021).

### 3.7 Renal and urinary system

#### 3.7.1 Bacteria

Urinary tract infections (UTIs) are commonplace afflictions, with culprits commonly including *E. coli*, *Proteus mirabilis*, *Klebsiella* spp., and *Staphylococcus saprophyticus*. These infections span across age groups, affecting both children and adults (Simões e Silva et al., 2019). UTI sequelae might present as clinical or scarring nephropathy, evident in 5% to 22% of affected individuals, typically emerging around six months post-infection resolution. Alarmingly, hypertension and chronic kidney disease might manifest as long-term repercussions from UTIs contracted during one’s younger years, presenting a daunting 15% risk of kidney failure subsequent to adolescence (San José Gonzáles and Méndez Fernández, 2009). Furthermore, a longitudinal study spanning 35 years revealed that an overwhelming 67% of women (n=58) who had experienced their first UTI eventually developed renal failure (Gebäck et al., 2015; Simões e Silva et al., 2019).

**Figure 8:**
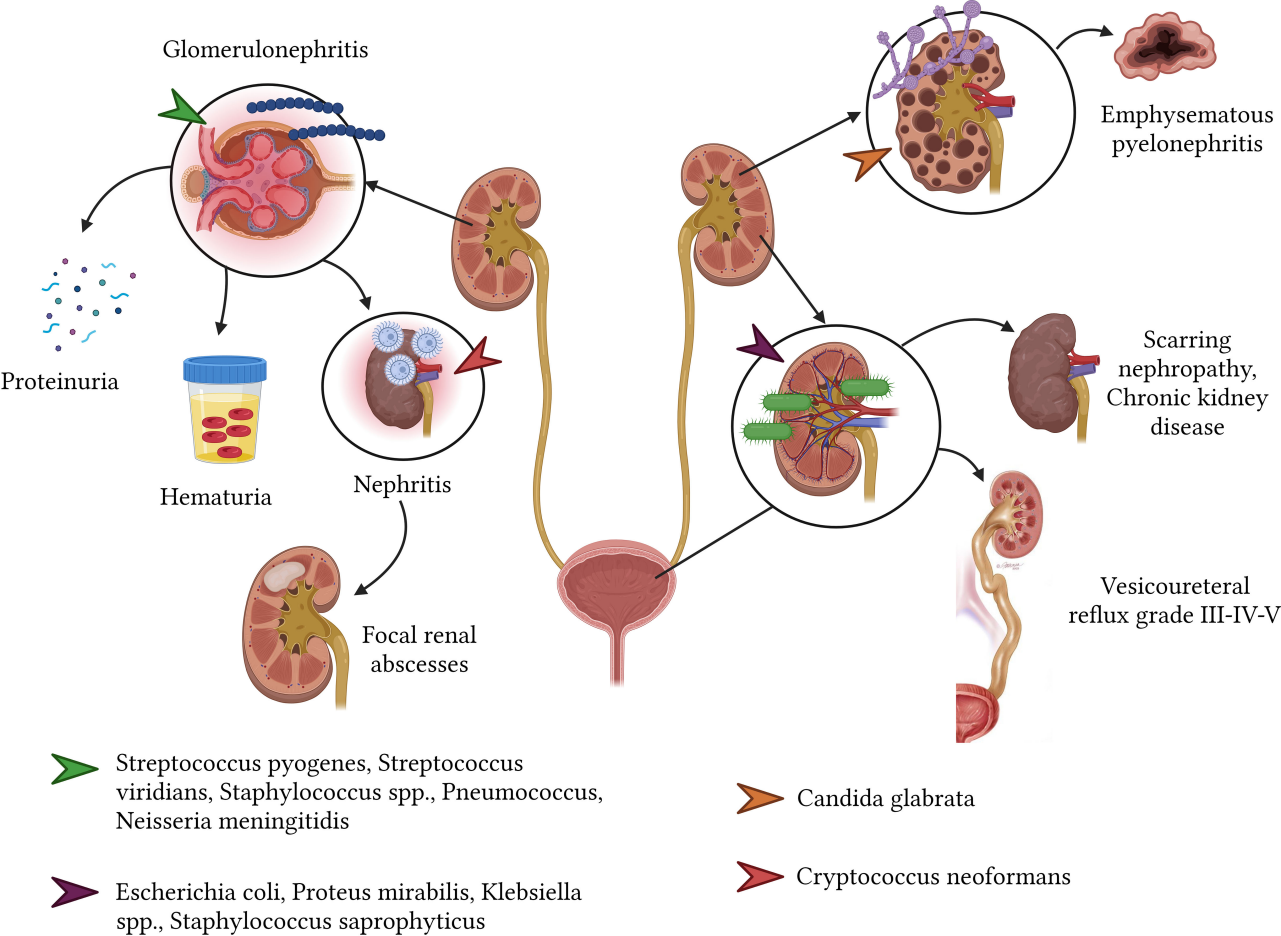
Sequelae of infectious diseases in the renal and urinary system.

Post-infectious glomerulonephritis (PIGN) stands out as a glomerular complication stemming from an immune reaction to an extrarenal infection. Overwhelmingly, more than 95% of PIGN cases correlate with *Streptococcus β hemolytic* group A, though other culprits like *Streptococcus viridians*, *Staphylococcus* spp., *S. pneumoniae*, and *N. meningitidis* are also identified. PIGN predominantly affects males, particularly those aged between 4 and 14 years. Potential sequelae encompass proteinuria (3.1%), microscopic hematuria (6.3%), and in rare instances linked with endocarditis concurrent with glomerulonephritis, post-infectious nephritis, showcasing IgA dominance and shunt nephritis (with an incidence of 27%) (Balasubramanian and Marks, 2017) (**Figure 8**).

#### 3.7.2 Virus

In the case of viral infections, recent research points toward the lingering ramifications of the SARS-CoV-2 virus on the renal system. Notably, post-30 days from an acute infection, survivors have exhibited an elevated risk of acute kidney injury (AKI). Furthermore, they displayed a diminished estimated glomerular filtration rate (eGFR). This adverse renal outcome’s likelihood escalated in direct proportion to the acute infection’s intensity (Bowe et al., 2021).

#### 3.7.3 Fungi

Disseminated coccidioidomycosis may involve the urinary tract, albeit rarely. The resulting sequelae largely depend on the location affected and the degree of acute infection-induced renal parenchymal destruction. Affected patients may develop progressive symptoms that vary over many months or years, including costovertebral angle pain, fatigue, urinary discomfort with or without fever, and the formation of genitourinary granulomas and abscesses (Coleman et al., 2021). In contrast, *Candida glabrata* can cause emphysematous pyelonephritis, potentially requiring total nephrectomy due to kidney loss (Schutz et al., 2022). Additionally, *C. neoformans* is associated with disseminated infections that may lead to pyelonephritis and the subsequent formation of focal renal abscesses (Wise, 2001).

### 3.8 Reproductive system

#### 3.8.1 Male

##### 3.8.1.1 Bacteria

Male genital tract infections, predominantly stemming from pathogens such as *C. trachomatis*, manifest acutely as urethritis, orchitis, epididymitis, and prostatitis (Dohle, 2003; Puerta-Suárez et al., 2014; Henkel, 2021). Left unchecked, these conditions can be detrimental to male fertility, leading to alterations in sperm attributes, including its morphology, viability, motility, and overall concentration. Elevated leukocyte presence within sperm has been associated with sperm weakening and subsequent genetic material degradation (Thorsen et al., 1991; Hosseinzadeh et al., 2000; Moazenchi et al., 2018; Henkel, 2021).

**Figure 9:**
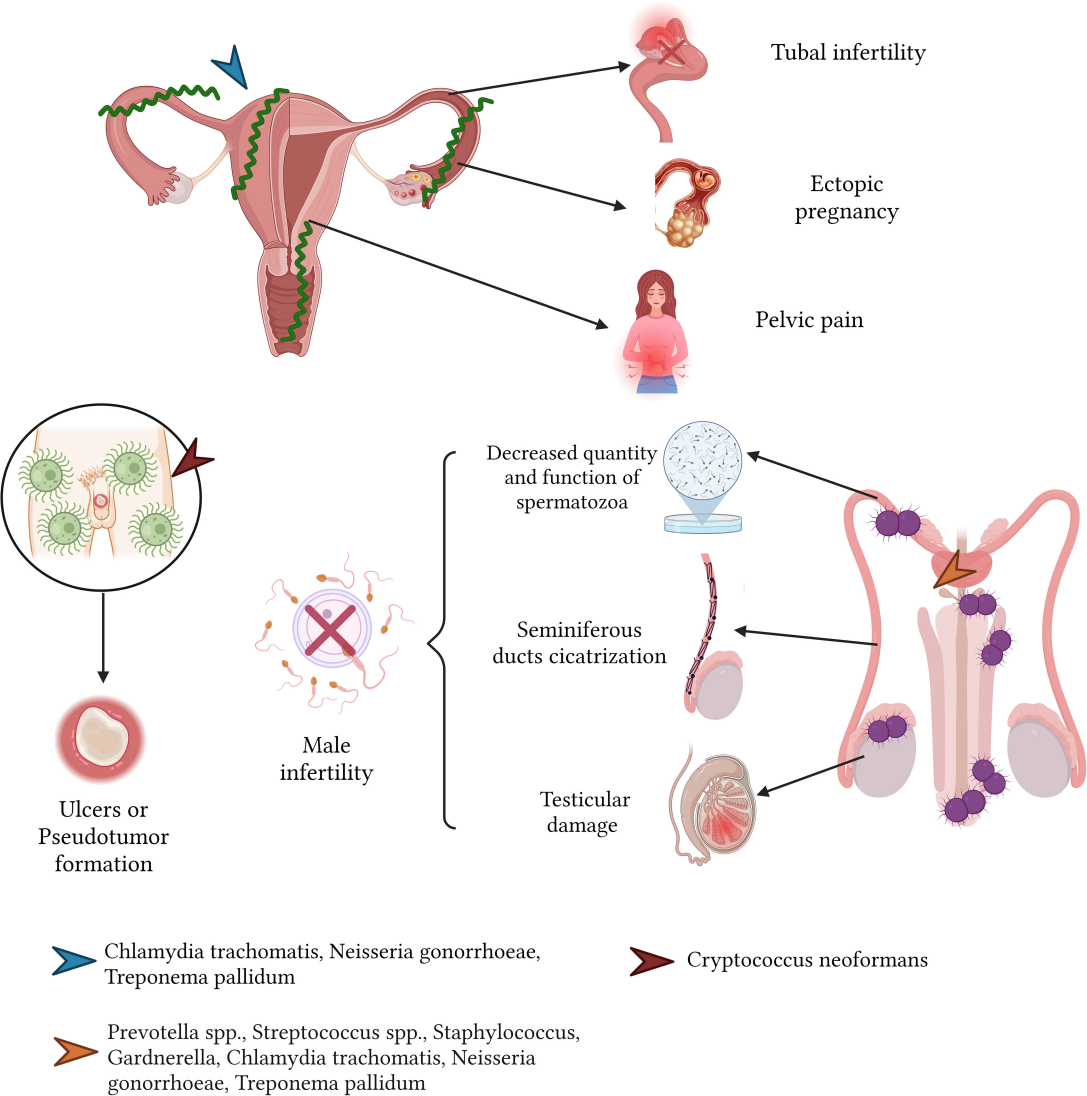
Sequelae of infectious diseases in the reproductive male and female system.

Similarly, infections from *N. gonorrhoeae* can induce urethritis, epididymitis, orchitis, and gonococcal prostatitis. The repercussions of these conditions include fertility challenges, as this bacterium can compromise sperm motility and viability. Additionally, scar tissue formation can block the seminiferous ducts, leading to conditions like obstructive azoospermia and subsequent testicular damage (Ochsner et al., 2016; Henkel, 2021) (**Figure 9**).

##### 3.8.1.2 Fungi

The *C. neoformans*, when affecting the male reproductive system—most commonly the penis—can lead to sequelae, typically in the form of isolated ulcers or pseudotumor formation (Wise, 2001).

#### 3.8.2 Female

##### 3.8.2.1 Bacteria

Bacterial vaginosis, precipitated by microbes such as *Gardnerella vaginalis*, *Ureaplasma urealyticum*, *Mycoplasma hominis*, and *Prevotella* spp., disrupts the harmonious balance of the vaginal environment. Such disturbances might attenuate sperm motility and instigate sperm mortality, culminating in fertility challenges (Ding et al., 2021; Ravel et al., 2021).

Sexually transmitted bacteria, including *C. trachomatis* and *N. gonorrhoeae*, are implicated in conditions such as acute pelvic inflammatory disease (PID) and endometritis. These ailments can spawn severe sequelae: fibrosis in the fallopian tubes, tubal infertility (affecting 15 to 20% of those with a PID history), chronic pelvic discomfort, and a heightened propensity for ectopic pregnancies (4 to 10 times the rate in the general female populace) (Deal et al., 2004; Kortekangas-Savolainen et al., 2012; Hafner, 2015) (**Figure 9**).

In the realm of obstetrics, bacterial vaginosis attributed to *G. vaginalis*, *U. urealyticum*, *M. hominis* can be the precursor to spontaneous miscarriages during the initial pregnancy trimester. As the pregnancy progresses, it can evoke premature membrane ruptures, early deliveries, and underweight newborns (Mania-Pramanik et al., 2009; Manns-James, 2011; Arif, 2018).

Intraamniotic bacterial infections, notably chorioamnionitis caused by pathogens like *L. monocytogenes*, can culminate in fetal demise and premature births (Kumar et al., 2022; Megli and Coyne, 2022). Concurrently, sexually transmitted infections due to *C. trachomatis* and *N. gonorrhoeae* are also associated with fetal losses, early membrane ruptures, preterm deliveries, and infants born underweight (Mårdh, 2002; Mullick et al., 2005).

##### 3.8.1.2 Fungi

Particularly, *Candida albicans*, among the Candida species, has been associated with the development of congenital cutaneous candidiasis and preterm deliveries (Mohamed et al., 2022).

## 4 Discussion

The immediate or acute stages of infectious diseases typically garner the majority of clinical attention due to their potentially life-threatening implications if untreated, including septicemia, sepsis, shock, and, ultimately, death. However, it is vital to acknowledge that the impact of infectious diseases often extends beyond their acute stages, often leading to enduring health consequences. While these consequences may not be immediately life-threatening, they pose considerable risks to health and quality of life and place a burden on healthcare systems. This has been starkly illustrated by the current COVID-19 pandemic, which has underscored the significant long-term health implications that can follow an infectious disease.

**Figure 10:**
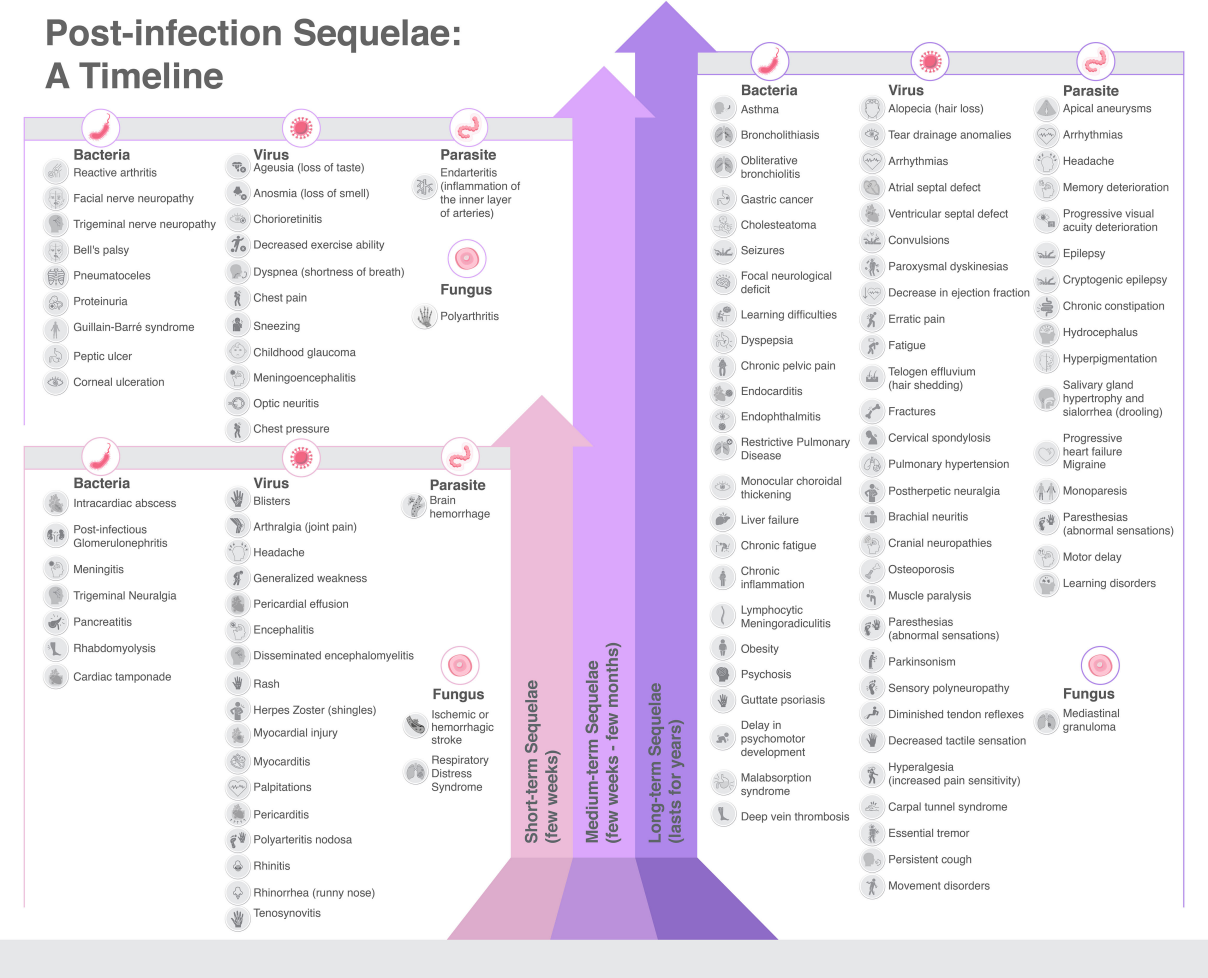
Sequelae caused by infectious diseases, categorized by etiology and time length of duration.

In our review, despite its narrative form, we have endeavored to explore, systematize, and catalog the multitude of sequelae that infectious diseases can engender, identifying a total of 274 sequelae. These are primarily induced by bacteria (38.3%) and viruses (31.0%), and less frequently by parasite (20.4%), and fungi (10.3%). Notably, bacteria such as *M. tuberculosis*, *S. aureus*, *E. coli*, and *S. pneumoniae*, alongside certain viruses, have been associated with high global mortality rates. Factors contributing to these mortality rates include gaps in vaccination programs, antibiotic resistance, and access barriers to antibiotic treatment in specific world regions (Institute for Health Metrics and Evaluation, 2019) (**Figure 10**).

Nonetheless, the long-term sequelae of infectious diseases play an integral role in this context. Many of these sequelae are classified as moderate (29.2%) or mild (26.3%), yet a substantial portion can cause disabling conditions (34.7%), such as stroke, liver failure, pigmentary retinopathy, Guillain Barre’s syndrome, and more. Some may even be life-threatening (9.9%), as demonstrated by diseases like gastric cancer, MALT lymphoma, pericardial effusion, and chagasic cardiomyopathy. Parasitic infections, followed by bacterial infections, represented the worst prognosis when assessing these sequelae (**Figure 10**). Furthermore, our review of sequelae severity has revealed that fungal infections do not typically result in very severe sequelae. Conversely, viral infections present a more evenly distributed range of mild (31.8%), moderate (30.6%), and severe (30.6%) sequelae. Bacterial infections are the predominant cause of sequelae, yet parasitic infections, mainly due to *T. cruzi*, pose a greater risk of serious sequelae, with 44.6% classified as severe and 21.4% as very severe (**Supplementary Table 1**).

The long-term effects or sequelae of infectious diseases can be significant and wide-ranging, impacting various body systems. From the examples provided in our text, it’s evident that infectious diseases can lead to chronic conditions and permanent changes in body function or structure. For instance, bacterial vaginosis can lead to infertility in women, and *C. trachomatis* and *N. gonorrhoeae* can cause Pelvic Inflammatory Disease, which may result in long-term complications such as tubal infertility and chronic pelvic pain. Similarly, intraamniotic bacterial infections can lead to fetal deaths and preterm deliveries. Hence, understanding these sequelae is essential for appropriate clinical management and disease prevention.

The study of post-acute consequences of infectious diseases remains limited. Chronic inflammation is posited as the primary mechanism for sequelae development in viral infections, compounded by the longer-than-anticipated persistence of viral particles. Additionally, it is speculated that subsequent coinfection with other viruses could contribute to aberrant innate immune signaling, further complicated by the emergence of autoantibodies from the adaptive immune system (Hirschenberger et al., 2021; Sette and Crotty, 2021).

The severity of the initial infection is a critical determinant in the progression of long-term effects. Episodes of severe sepsis have been correlated with poorer long-term outcomes, resulting in cognitive and functional impairments in patients. The underpinning mechanisms, however, have yet to be fully elucidated. It is uncertain whether the long-term consequences in severe cases are directly linked to the pathogenic microorganism, polymicrobial infections, or resistant organisms (Leibovici, 2013). This uncertainty likely stems from the fact that most exploratory studies have not extended their assessment of the sequelae of septic conditions beyond six weeks.

Although our review did not encompass vital aspects such as the affected age groups or antimicrobial resistance, it underscores the necessity of further research into the long-term outcomes of infectious diseases. Beyond basic biomedical research, we advocate for the refinement of diagnostic criteria, the establishment of a unified nomenclature, and the acquisition of more reliable prevalence and social burden data related to the sequelae of infectious diseases (Choutka et al., 2022).

Healthcare systems globally are already burdened with the acute management of infectious diseases, but the expenses do not stop at the acute phase. Long-term health sequelae can require years of ongoing care, sometimes lasting a lifetime, and add considerable costs to health systems. For example, the residual effects of polio such as leg deformities can necessitate lifelong mobility aids and physical therapy. Similarly, the persistent “brain fog” reported by many COVID-19 survivors can impair occupational functioning, leading to productivity loss and long-term disability. Reproductive sequelae, such as uterine scarring following pelvic infections, can lead to fertility issues requiring expensive treatments or interventions.

Despite these impacts the focus on these long-term consequences is disproportionately low relative to their socio-economic implications. Unfortunately, this comparative lack of awareness means that opportunities for early intervention, which could potentially mitigate these outcomes, are frequently overlooked. These long-term health effects are not merely an extension of the initial infectious disease but represent a new phase of illness that demands our attention and resources.

In the wake of COVID-19’s long-term effects, now a significant public health challenge, it is imperative to recognize that many other infectious diseases could have similar enduring health implications, which may be neglected. We hope that this review not only serves as an informational aid for clinicians endeavoring to understand the long-term effects of infectious diseases but also as a clarion call to scientists and health authorities. This facet of global health requires concentrated attention and devoted resources for both prevention and management.

### 4.1 Limitations

This narrative review carries inherent limitations that warrant discussion. Despite our extensive efforts to cover a broad range of infectious disease sequelae, the absence of a standardized methodological framework characteristic of structured literature reviews means our study may bear potential bias in the selection, analysis, and interpretation of the literature incorporated. As a result, this could lead to an inadvertent overemphasis or under-representation of certain infectious diseases and their respective sequelae. Furthermore, our discussion may not fully capture the complex interactions between host factors, infection characteristics, and societal determinants that contribute to the diversity of sequelae observed in different populations.

We acknowledge that the conclusions drawn herein are qualitative in nature and, while they provide important insights, may not deliver precise quantitative estimates of the incidence, prevalence, or impact of the various sequelae. To obtain more robust, statistically supported insights into the long-term impacts of infectious diseases, further research is needed. We suggest that subsequent systematic reviews or meta-analyses be conducted, complemented by cohort studies where feasible, to provide a more comprehensive and quantitatively reliable understanding of these significant health consequences. These future studies would ideally follow a rigorous protocol for literature selection, data extraction, and synthesis to minimize bias and maximize the applicability of the findings.

### 4.2 Future directions and conclusions

Determining the cause-and-effect relationship between infectious diseases and their ensuing long-term sequelae is a methodological challenge, yet it remains a cornerstone for scientific advancement. Specifically, unravelling these causal links can pave the way to uncover novel molecular mechanisms, subsequently opening avenues for the development of new interventions or the repurposing of existing therapies. Techniques such as Mendelian randomization in the realm of host genetics can be instrumental in mitigating confounding factors, thereby leading to more accurate and reliable results. However, comprehensive efforts to define these mechanistic relationships need to be intensified, combining epidemiological studies, host genetics, and laboratory research.

Acknowledging the distinct phases of various infectious diseases could enable future research to discern the specific impact of the diseases and their unique pathogenic pathways on the development of sequelae. The overall impact likely stems from a combination of impaired host resistance, deficient tolerance, and the general effect of frailty and reduced functional reserve. In this context, public health efforts geared toward bolstering baseline population health emerge as an integral component of preparedness against the long-term implications of infectious diseases.

The substantial burden of disease generated by the sequelae of infectious diseases, particularly when considering conditions like post-acute sequelae of SARS-CoV-2 infection (PASC) and the broader public health implications of infectious diseases, warrants urgent attention. The overlay of infectious disease sequelae on top of the existing widespread non-communicable disease comorbidities and socioeconomic inequality forms a ‘syndemic’ that poses significant public health challenges.

Considering the continued evolution of various pathogens and the potential for co-infections, coupled with changes in baseline host resistance due to factors like vaccination, the patterns of disease and their sequelae will undoubtedly continue to evolve. This evolution underscores the pressing need to maintain research focus on the significant influence of infectious diseases on long-term health outcomes. Through continued vigilance, research, and investment, we can hope to mitigate the burden of these diseases and their sequelae on the global healthcare systems, thereby improving the quality of life for those affected.

## Author contributions

JI: Conceptualization, Data curation, Formal analysis, Investigation, Methodology, Project administration, Resources, Visualization, Writing – original draft, Writing – review & editing. JV: Investigation, Methodology, Software, Validation, Visualization, Writing – original draft. EM: Data curation, Formal analysis, Investigation, Methodology, Resources, Software, Validation, Visualization, Writing – original draft. AT: Data curation, Investigation, Resources, Software, Validation, Visualization, Writing – original draft. PN: Data curation, Investigation, Methodology, Resources, Software, Visualization, Writing – original draft. RF: Investigation, Methodology, Resources, Validation, Visualization, Writing – original draft. MH: Data curation, Investigation, Methodology, Resources, Software, Visualization, Writing – original draft. AE: Formal analysis, Investigation, Methodology, Resources, Visualization, Writing – original draft. VY: Formal analysis, Investigation, Methodology, Resources, Software, Visualization, Writing – original draft. AD: Investigation, Methodology, Resources, Software, Visualization, Writing – original draft. CO: Data curation, Investigation, Methodology, Resources, Visualization, Writing – original draft. EO: Conceptualization, Investigation, Methodology, Project administration, Resources, Supervision, Validation, Visualization, Writing – review & editing.
