# Supplementary material for: Beyond the acute phase: a comprehensive literature review of long-term sequelae resulting from infectious diseases
Source: Front Cell Infect Microbiol. 2024 Jan 31;14:1293782. doi: 10.3389/fcimb.2024.1293782 (PMC10864624; doi:10.3389/fcimb.2024.1293782)
Supplement: Supplementary file 1 [file DataSheet_1.docx]

**Supplementary table 1.** Summary of the sequelae caused by infectious diseases according to their origin and severity.

| **Sequelae severity** | **Sequelae etiology** | | | | | | | |
| --- | --- | --- | --- | --- | --- | --- | --- | --- |
|  | **Bacteria** | | **Virus** | | **Parasite** | | **Fungi** | |
| **Mild** | Driving abnormalities | *Methicillin-resistant S. aureus (MRSA), S. pyogenes, S. pneumoniae, C. burnetti, N. gonorrhoeae* | Ageusia | SARS-CoV-2 | Headache | *T. gondii* | Permanent recalcitrant scars | *T. rubrum, T. mentagrophytes, E. floccosum* |
|  | Asthma | *S. pneumoniae* | Alopecia | SARS-CoV-2 | Permanent scarring | *Leishmania* | Mediastinal granuloma | *H. capsulatum* |
|  | Ataxia | *H. influenzae* | Blisters | SARS-CoV-2 | Onychodystrophy | *T. penetrans, T. trimamillata* | Pustular acneiform lesions | *C. immitis, C. posadasii* |
|  | Scarring atelectasis | *M. tuberculosis* | Anosmia | SARS-CoV-2 | Endarteritis | *T. solium* | Ulcerated and warty plaques | *C. immitis, C. posadasii* |
|  | Low birth weight | *C. trachomatis, N. gonorrhoeae, T. pallidum* | Arthralgia | Rubivirus rubellae | Salivary gland hypertrophy and sialorrhea | *T. cruzi* | Pain in the costovertebral angle | *C. immitis, C. posadasii* |
|  | Broncholithiasis | *M. tuberculosis* | Headache | SARS-CoV-2, HIV | Monoparesis | *P. falciparum* | Fatigue | *C. immitis, C. posadasii* |
|  | Bronchiectasis | *H. influenzae, S. pneumoniae, M. catarrhalis, S. aureus, P. aeruginosa, M. tuberculosis* | Generalized weakness | SARS-CoV-2 | Paresthesias | *Echinococcus* | Urinary discomfort with or without fever | *C. immitis, C. posadasii* |
|  | Scarring of the seminiferous ducts | *N. gonorrheae* | Decreased DLCO | SARS-CoV-2, MERS-CoV, SARS-CoV | Loss of toenails | *T. penetrans, T. trimamillata* | Pulmonary nodules | *C. immitis, C. posadasii* |
|  | Testicular damage | *N. gonorrheae* | Decreased ability to be physically active | MERS-CoV, SARS-CoV |  |  | Pulmonary cavities | *C. immitis, C. posadasii* |
|  | Diabetes insipidus | *M. tuberculosis* | Dyspnea | SARS-CoV-2 |  |  |  |  |
|  | Decreased sperm count and functionality | *C. trachomatis, N. gonorrhoeae* | Chest pain | SARS-CoV-2 |  |  |  |  |
|  | Dyspepsia | *H. pylori, C. jejuni, C. coli, Salmonella spp, Y. enterocolitica, Shigella spp, E. coli* | Erratic pain | SARS-CoV-2 |  |  |  |  |
|  | Ectopic pregnancy | *C. trachomatis, N. gonorrhoeae* | Telogen effluvium | SARS-CoV-2 |  |  |  |  |
|  | Pleural thickening | *M. tuberculosis* | Sneezing | SARS-CoV-2 |  |  |  |  |
|  | Strabismus | *S. pneumoniae* | Exanthema | SARS-CoV-2 |  |  |  |  |
|  | Chronic fatigue | *MRSA, S. pyogenes, S. pneumoniae* | Fatigue | SARS-CoV-2, MERS-CoV, SARS-CoV |  |  |  |  |
|  | Mediastinal fibrosis | *M. tuberculosis* | Chest tightness | SARS-CoV-2 |  |  |  |  |
|  | Hematuria | *S. pyogenes* | Palpitations | SARS-CoV-2 |  |  |  |  |
|  | High blood pressure | *E. coli, P. mirabilis, Klebsiella spp., S. saprophyticus* | Paresthesias | SARS-CoV-2 |  |  |  |  |
|  | Male infertility | *C. trachomatis, N. gonorrhoeae* | Hair loss | SARS-CoV-2 |  |  |  |  |
|  | Tubal infertility | *C. trachomatis, N. gonorrhoeae* | Diminished tendon reflexes | SARS-CoV-2 |  |  |  |  |
|  | Chronic inflammation | *H. Pylori* | Rhinitis | SARS-CoV-2 |  |  |  |  |
|  | Paresis | *H. influenzae* | Rhinorrhea | SARS-CoV-2 |  |  |  |  |
|  | Pneumatoceles | *S. aureus* | Diminished tactile sensation | SARS-CoV-2 |  |  |  |  |
|  | Proteinuria | *S. pyogenes* | Hyperalgesia | SARS-CoV-2 |  |  |  |  |
|  | Guttate psoriasis | *S. pyogenes* | Essential tremor | SARS-CoV-2 |  |  |  |  |
|  | Peptic ulcer | *H. pylori* | Persistent cough | SARS-CoV-2 |  |  |  |  |
| **Moderate** | Intracardiac abscess | *S. aureus* | Tear drainage abnormalities | Rubivirus rubellae | Cardiac conduction abnormalities | *T. cruzi* | Chronic fibrocavitary pneumonia | *C. immitis, C. posadasii* |
|  | Behavioral disturbances | *S. pneumoniae* | Arrhythmias | DENV-1, DENV-2, DENV-3, DENV-4, Chikungunya virus | Arrhythmias | *T. cruzi* | Permanent alopecia | *T. schönleinii* |
|  | Mental disturbances | *M. tuberculosis* | Falls | Rubivirus rubellae | Nerve entrapment | *T. solium* | Angina | *H. capsulatum* |
|  | Reactive arthritis | *C. trachomatis, Salmonella, Shigella, Campylobacter, Yersinia* | Seizures | HIV | Seizures | *P. falciparum, T. solium* | Bilateral visual impairment | *C. neoformans gatti variant* |
|  | Intracranial calcifications | *M. tuberculosis* | Paroxysmal dyskinesias | HIV | Visual field defects | *T. solium* | Polyarthritis | *H. capsulatum* |
|  | Ulcerative colitis | *C. jejuni, Salmonella no tifoidea spp, Y. enterocolitica, Shigella spp, E. coli enterohemorrágica, Listeria* | Myocardial edema | SARS-CoV-2 | Chronic constipation | *T. cruzi* | Respiratory Distress Syndrome | *Blastomyces dermatitidis* |
|  | Seizures | *E. coli, S. agalactiae, L. monocytogenes, H. influenzae, S. pneumoniae* | Cervical spondylosis | Wild Poliovirus type 1, tipe 2, type 3 | Hemiparesis | *T. gondii* | Permanent abscesses and fistulas | *C. immitis, C. posadasii* |
|  | Focal neurological deficit | *S. pneumoniae* | Peripheral pulmonary artery stenosis | Rubivirus rubellae | Hydrocephalus | *T. solium* | Bone cysts | *C. immitis, C. posadasii* |
|  | Chronic pelvic pain | *C. trachomatis, N. gonorrhoeae* | Aortic valve stenosis | Rubivirus rubellae | Migraine | *T. gondii* | Chronic back pain | *Candida spp.* |
|  | Crohn's disease | *C. jejuni, Salmonella no tifoidea spp, Y. enterocolitica, Shigella spp, E. coli enterohemorrágica, Listeria* | Pulmonary valve stenosis | Rubivirus rubellae | Learning disabilities | *T. gondii* | Nerve compression Syndrome | *Candida spp.* |
|  | Restrictive lung disease | *S. pneumoniae, H. influenzae* | Pulmonary fibrosis | SARS-CoV-2 |  |  | Genitourinary granulomas | *C. immitis, C. posadasii* |
|  | Hepatic artery stenosis | *K. pneumoniae, E. coli, Enterobacter spp., Proteus spp., Pseudomona spp.* | Fractures | Wild Poliovirus type 1, tipe 2, type 3 |  |  | Kidney abscesses | *C. immitis, C. posadasii* |
|  | Hydrocephalus | *E. coli, S. agalactiae, L. monocytogenes, H. influenzae, S. pneumoniae* | Shingles | Varicella-zoster virus |  |  | Focal Renal Abscesses | *C. neoformans* |
|  | Recurrent infections | *M. tuberculosis* | Pulmonary hypertension | HIV |  |  | Penile ulcers | *C. neoformans* |
|  | IgA-predominant infectious nephritis | *S. pyogenes, S. viridians, Staphylococcus spp., S. pneumoniae, N. meningitidis* | Lateral instability of the knee | Wild Poliovirus type 1 |  |  | Penile pseudotumors | *C. neoformans* |
|  | Trigeminal neuralgia | *B. burgdorferi* | Axonal mononeuropathies | SARS-CoV-2 |  |  |  |  |
|  | Facial nerve neuropathy | *B. burgdorferi* | Postherpetic neuralgia | Varicella-zoster virus |  |  |  |  |
|  | Trigeminal nerve neuropathy | *B. burgdorferi* | Brachial neuritis | HIV type 1 |  |  |  |  |
|  | Vestibulocochlear nerve neuropathy | *B. burgdorferi* | Osteoarthritis | Wild Poliovirus type 1 |  |  |  |  |
|  | Obesity | *M. tuberculosis* | Osteoporosis | Wild Poliovirus type 1 |  |  |  |  |
|  | Preterm delivery | *G. vaginalis, U. urealyticum, M. hominis, L. monocytogenes* | Parkinsonism | SARS-CoV-2 |  |  |  |  |
|  | Loss of lung parenchyma | *M. tuberculosis* | Polyarteritis nodosa | VHB |  |  |  |  |
|  | Rhabdomyolysis | *K. pneumoniae, E. coli, E. spp., Proteus spp. y Pseudomonas spp.* | Sensory polyneuropathy | HIV type 1 |  |  |  |  |
|  | Reduced visual acuity | *C. trachomatis* | Carpal tunnel syndrome | Rubivirus rubellae |  |  |  |  |
|  | Delayed psychomotor development | *E. coli, S. agalactiae, L. monocytogenes, H. influenzae, S. pneumoniae* | Tenosynovitis | Rubivirus rubellae |  |  |  |  |
|  | Premature rupture of membranes | *G. vaginalis, U. urealyticum, M. hominis, C. trachomatis, N. gonorrhoeae* |  |  |  |  |  |  |
|  | Malabsorption syndrome | *C. jejuni, C. coli, Salmonella no tifoidea spp., Y. enterocolitica, Shigella spp., E. coli enterohemorrágica, Listeria spp.* |  |  |  |  |  |  |
|  | Irritable bowel syndrome | *C. jejuni, C. coli, Salmonella no tifoidea spp., Y. enterocolitica, Shigella spp., E. coli enterohemorrágica, Listeria spp.* |  |  |  |  |  |  |
|  | Corneal ulceration | *N. gonorrhoeae* |  |  |  |  |  |  |
| **Severe** | Stroke | *MRSA, S. pyogenes, S. pneumoniae, C. burnetti, N. gonorrhoeae* | Dilated cardiomyopathy | Chikungunya virus | Apical aneurysms | *T. cruzi* | Dilated cardiomyopathy | *Candida spp.* |
|  | Shortening of the limb | *MRSA, S. pyogenes, S. pneumoniae y Enterobacter spp.* | Atrial septal defect | Rubivirus rubellae | Speech abnormality | *T. gondii* | Necrosis of perivascular and intravascular tissues | *Zygomycetes* |
|  | Amputation | *MRSA, S. pyogenes, S. pneumoniae y Enterobacter spp.* | Ventricular septal defect | Rubivirus rubellae | Brain atrophy | *T. solium* | Total nephrectomy | *C. glabatra* |
|  | Rasmussen's aneurysms | *M. tuberculosis* | Chorioretinitis | Rubivirus rubellae | Self-amputation of the fingers | *T. penetrans, T. trimamillata* | Lytic bone lesions | *C. immitis, C. posadasii* |
|  | Bronchiolitis obliterans | *M. pneumoniae* | Cloudy cornea | Rubivirus rubellae | Left anterior fascicular block | *T. cruzi* |  |  |
|  | Blindness | *N. gonorrhoeae* | Fixed flexion deformities | Wild Poliovirus type 1 | Cardiomegaly | *T. cruzi* |  |  |
|  | Monocular blindness | *N. gonorrhoeae* | Disability | Wild Poliovirus type 1, tipe 2, type 3 | Partial or complete destruction of the mucous membranes of the mouth | *Leishmania* |  |  |
|  | Kidney scarring | *E. coli, P. mirabilis, Klebsiella spp., S. saprophyticus* | Cognitive dysfunction | HIV type 1 | Partial or complete destruction of the mucous membranes of the nose | *Leishmania* |  |  |
|  | Corneal scarring with loss of corneal transparency | *C. trachomatis* | Decreased ejection fraction | SARS-CoV-2 | Memory impairment | *S. haematobium, P. falciparum* |  |  |
|  | Cholesteatoma | *S. pneumoniae, H. influenzae, M. catarrhalis* | Encephalitis | Varicella-zoster virus, Rubivirus rubellae, Herpes virus types 6 and 7, West Nile virus, Enterovirus, Epstein-Barr virus, Cytomegalovirus, Measles, Mumps virus, St. Louis virus, Eastern equine virus, Western equine virus, Dengue virus, Rabies virus, VHB, VHC | Progressive deterioration of visual acuity | *T. solium* |  |  |
|  | Vestibular damage | *H. influenzae* | Scoliosis | Wild Poliovirus type 1 | Difficulty walking | *T. penetrans, T. trimamillata* |  |  |
|  | Severe destruction of the joint | *MRSA, S. pyogenes, S. pneumoniae y Enterobacter spp.* | Infantile glaucoma | Rubivirus rubellae | Speech disability | *P. falciparum* |  |  |
|  | Cognitive impairment | *S. pneumoniae* | Pulmonary artery hypoplasia | Rubivirus rubellae | Intellectual disability | *T. gondii* |  |  |
|  | Learning disability | *H. influenzae* | Iris hypoplasia | Rubivirus rubellae | Visual impairment | *P. falciparum* |  |  |
|  | Ossicular discontinuity | *S. pneumoniae, H. influenzae, M. catarrhalis* | Myocardial injury | Chikungunya virus | Epilepsy | *T. solium, T. gondii* |  |  |
|  | Embolization without stroke | *MRSA, S. pyogenes, S. pneumoniae, C. burnetti, N. gonorrhoeae* | Heart failure | Chikungunya virus, Dengue virus | Cryptogenic epilepsy | *T. gondii* |  |  |
|  | Endophthalmitis | *K. pneumoniae, E. coli, Enterobacter spp., Proteus spp. Pseudomonas spp.* | Brain Injuries | MERS-CoV, SARS-CoV | Schizophrenia | *T. gondii* |  |  |
|  | Chronic kidney disease | *E. coli, P. mirabilis, Klebsiella spp., S. saprophyticus* | Reticular or cystic lesions | SARS-CoV-2 | Endomyocardial fibrosis | *T. cruzi* |  |  |
|  | Rheumatic heart disease | *S. pyogenes* | Meningoencephalitis | HIV type 1 | Severe liver fibrosis | *T. cruzi* |  |  |
|  | Monocular choroidal thickening | *N. gonorrhoeae* | Microphthalmia | Rubivirus rubellae | Progressive heart failure | *T. cruzi* |  |  |
|  | Liver failure | *C. jejuni, C. coli, Salmonella no tifoidea spp., Y. enterocolitica, Shigella spp., E. coli enterohemorrágica, Listeria spp.* | Myelopathy | HIV type 1 | Paraplegia | *Echinococcus* |  |  |
|  | Ossicular fixation | *S. pneumoniae, H. influenzae, M. catarrhalis* | Myocarditis | Chikungunya virus, Dengue virus | Hearing loss | *T. gondii* |  |  |
|  | Post-infectious glomerulonephritis | *S. pyogenes, S. viridians, Staphylococcus spp., S. pneumoneae, N. meningitidis* | Optic neuritis | HIV type 1 | Psychomotor delay | *T. gondii* |  |  |
|  | Spastic hemiplegia | *H. influenzae* | Cranial neuropathies | HIV type 1 | Brown-Séquard syndrome | *Echinococcus* |  |  |
|  | Conduction and sensorineural hearing loss | *S. pneumoniae, H. influenzae, M. catarrhalis* | Retinitis pigmentosa | Rubivirus rubellae | Thrombosis | *T. solium, S. mansoni, S. haematobium, S. japonicum* |  |  |
|  | Heart failure | *S. pyogenes* | Ischemic or hemorrhagic stroke | Varicella-zoster virus |  |  |  |  |
|  | Upper and lower motor neuron injury | *M. tuberculosis* |  |  |  |  |  |  |
|  | Lymphocytic meningoradiculitis (Bannwarth syndrome) | *B. burgdorferi* |  |  |  |  |  |  |
|  | Dilated cardiomyopathy | *S. aureus, S. pyogenes, S. pneumoniae, N. menigitis, H. influenzae, C. diphtheriae, M. pneumoniae, C. Burnetti, T. pallidum, Borrelia, Leptospira* |  |  |  |  |  |  |
|  | Joint necrosis | *MRSA, S. pyogenes, S. pneumoniae y Enterobacter spp.* |  |  |  |  |  |  |
|  | Corneal neovascularization | *C. trachomatis, N. gonorrhoeae* |  |  |  |  |  |  |
|  | Neurosyphilis | *T. pallidum* |  |  |  |  |  |  |
|  | Adhesive otitis media | *S. pneumoniae, H. influenzae, M. catarrhalis* |  |  |  |  |  |  |
|  | Bell's palsy | *B. burgdorferi* |  |  |  |  |  |  |
|  | Paraplegia | *M. tuberculosis* |  |  |  |  |  |  |
|  | Hearing loss | *S. pneumoniae, N. meningitidis* |  |  |  |  |  |  |
|  | Psychosis | *H. influenzae* |  |  |  |  |  |  |
|  | Tympanosclerosis | *S. pneumoniae, H. influenzae, M. catarrhalis* |  |  |  |  |  |  |
|  | Severe growth disorder | *MRSA, S. pyogenes, S. pneumoniae y Enterobacter spp.* |  |  |  |  |  |  |
|  | Deep vein thrombosis | *MRSA, S. pyogenes, S. pneumoniae y Enterobacter spp.* |  |  |  |  |  |  |
| **Very severe** | Gastric cancer | *H. pylori* | Movement disorders | HIV type 1 | Right bundle branch block | *T. cruzi* |  |  |
|  | Pulmonary embolism | *MRSA, S. pyogenes, S. pneumoniae y Enterobacter spp.* | Coarctation of the aorta | Rubivirus rubellae | Brain hemorrhage | *S. mansoni, S. haematobium, S. japonicum* |  |  |
|  | Endocarditis | *MRSA, S. pyogenes, S. pneumoniae, C. Burnetti, N. gonorrhoeae* | Pericardial effusion | Dengue virus | Hemorrhagic or ischemic stroke | *T. solium* |  |  |
|  | MALT lymphoma | *H. pylori* | Disseminated encephalomyelitis | HIV type 1 | Megacoledochus | *T. cruzi* |  |  |
|  | Meningitis | *K. pneumoniae, E. coli, Enterobacter spp., Proteus spp., Pseudomona spp.* | Muscle paralysis | Wild Poliovirus type 1, tipe 2, type 3 | Megacolon | *T. cruzi* |  |  |
|  | Pancreatitis | *C. jejuni, C. coli, Salmonella no tifoidea spp., Y. enterocolitica, Shigella spp., E. coli enterohemorrágica, Listeria spp.* | Pericarditis | Dengue virus | Megaduodenum | *T. cruzi* |  |  |
|  | Constrictive pericarditis | *M. tuberculosis, S. pneumoniae, H. influenzae, M. pneumoniae, C. burnetii, B. burgdoreri* | Tetralogy of Fallot | Rubivirus rubellae | Megaesophagus | *T. cruzi* |  |  |
|  | Guillain-Barré syndrome | *C. jejuni, C. coli, Salmonella no tifoidea spp., Y. enterocolitica, Shigella spp., E. coli enterohemorrágica, Listeria spp.* |  |  | Megastomach | *T. cruzi* |  |  |
|  | Cardiac tamponade | *M. tuberculosis* |  |  | Megagallbladder | *T. cruzi* |  |  |
|  |  |  |  |  | Megajejunum | *T. cruzi* |  |  |
|  |  |  |  |  | Chronic Chagasic cardiomyopathy | *T. cruzi* |  |  |
|  |  |  |  |  | Fibrosing myocarditis | *T. cruzi* |  |  |

**Supplementary table 2.** Summary of the evidence on sequelae attributed to infectious diseases.

| **Pathogen** | **System/Tissue affected** | **Type of study** | **Reference** |
| --- | --- | --- | --- |
| *Escherichia coli* | Nervous System | Descriptive | Olmedo D et al. (Olmedo Díaz et al., 1997) |
|  |  | Review | Suthar R. (Suthar and Sankhyan, 2019) |
|  | Gastrointestinal Tract | Review | Pogreba-Brown K. et al.(Pogreba-Brown et al., 2020) |
|  | Renal and Urinary System | Review | Simões e Silva A. et al. (Simões e Silva et al., 2019) |
| *Group B β-hemolytic Streptococcus* | Nervous System | Descriptive | Olmedo D. et al. (Olmedo Díaz et al., 1997) |
|  |  | Review | Suthar R. (Suthar and Sankhyan, 2019) |
| *Streptococcus agalactiae* | Nervous System | Review | Suthar R. (Suthar and Sankhyan, 2019) |
| *Listeria monocytogenes* | Nervous System | Descriptive | Olmedo D. et al. (Olmedo Díaz et al., 1997) |
|  |  | Review | Suthar (Suthar and Sankhyan, 2019) |
| *Haemophilus influenzae* | Nervous System | Review | Suthar R. (Suthar and Sankhyan, 2019), Lucas MJ. et al. (Lucas et al., 2016) |
|  | Cardiovascular System | Book | Cecchini E. (Cecchini and González Ayala, 2011) |
|  | Respiratory System | Review | Pizzutto S. et al. (Pizzutto et al., 2017), Edmond K. et al. (Edmond et al., 2012) |
|  |  | Book | Martinez-Pitre P. et al. (Martinez-Pitre et al., 2022) |
| *Streptococcus pneumoniae* | Nervous System | Review | Lucas M. et al. (Lucas et al., 2016) |
|  | Cardiovascular System | Book | Cecchini E. (Cecchini and González Ayala, 2011) |
|  | Respiratory System | Review | Pizzutto S. et al. (Pizzutto et al., 2017), Edmond K. et al. (Edmond et al., 2012), Grimwood K. (Grimwood and Chang, 2015) |
|  |  | Book | Martinez-Pitre P. et al. (Martinez-Pitre et al., 2022) |
|  |  | Cohort | Rhedin S. et al. (Rhedin et al., 2022) |
|  | Musculoskeletal System | Review | Godley D. (Godley, 2015) |
|  |  | Research | Saeed K. et al. (Saeed et al., 2021) |
|  |  | Book | Ochsner P. et al. (Ochsner et al., 2016) |
| *Neisseria meningitidis* | Nervous System | Review | Lucas M. et al. (Lucas et al., 2016) |
|  | Renal and Urinary System | Review | Balasubramanian R. (Balasubramanian and Marks, 2017) |
| *Mycobacterium tuberculosis* | Nervous System | Case report | Palacio Sanguino A. (Palacio Sanguino and Arteaga, 2020) |
|  |  | Descriptive | Todd R. (Todd and Neville, 1964), Kennedy D. (Kennedy and Fallon, 1979), Lorber J. (Lorber, 1958) |
|  | Cardiovascular System | Book | Cecchini E. (Cecchini and González Ayala, 2011) |
|  | Respiratory System | Review | Romero Marín et al. (Romero Marín et al., 2016), Kim H. et al. (Kim et al., 2001) |
|  |  | Observational | Aggarwal N. et al. (Aggarwal et al., 2021) |
| *Treponema pallidum* | Nervous System | Review | Ropper A. (Ropper, 2019) |
|  |  | Book | Bhandari J. et al. (Bhandari et al., 2022) |
|  |  | Case report | Nassar Tobón A. et al. (Nassar Tobón et al., 2020) |
| *Borrelia burgdorferi* | Nervous System | Review | Brizzi K. (Brizzi and Lyons, 2014), Halperin J. (Halperin, 2003), Halperin J. (Halperin, 2016) |
| *Chlamydia trachomatis* | Eyes | Book | Graue E. (Graue Wiechers, 2014) |
|  |  | Review | Mullick S. et al. (Mullick et al., 2005), Mårdh P. (Mårdh, 2002) |
|  | Reproductive System | Review | Henkel R. (Henkel, n.d.), Puerta-Suárez J. et al. (Puerta-Suárez et al., 2014), Hafner L. (Hafner, 2015), Deal C. et al. (Deal et al., 2004) |
|  |  | Cohort | Dohle G. (Dohle, 2003), Hosseinzadeh S. et al. (Hosseinzadeh et al., 2000), Thorsen P. et al. (Thorsen et al., 1991), Moazenchi M. et al. (Moazenchi et al., 2018), Mania-Pramanik J. et al. (Mania-Pramanik et al., 2009) |
|  |  | Retrospective | Kortekangas-Savolainen O. et al. (Kortekangas-Savolainen et al., 2012) |
| *Neisseria gonorrhoeae* | Ears | Review | Arellano Barriga G. et al. (Arellano Barriga et al., 2020), Mullick S. et al. (Mullick et al., 2005) |
|  |  | Book | Graue E. (Graue Wiechers, 2014) |
|  | Eyes | Case report | Turati M. (Turati and Turati, 2000), Petour F. et al. (Petour et al., 2016) |
|  | Cardiovascular System | Book | Cecchini E. (Cecchini and González Ayala, 2011) |
|  | Reproductive System | Review | Henkel R. (Henkel, n.d.) |
| *Moraxella catarrhalis* | Ears | Review | Bluestone C. (Bluestone, 2000) |
|  | Respiratory System | Review | Pizzutto S. (Pizzutto et al., 2017) |
| *Staphylococcus aureus* | Cardiovascular System | Book | Cecchini E. (Cecchini and González Ayala, 2011) |
|  |  | Review | Vincent L. (Vincent and Otto, 2018) |
|  | Respiratory System | Review | Daltro P. et al. (Daltro et al., 2011), Pizzutto S. et al. (Pizzutto et al., 2017) |
|  | Integumentary System | Review | Chiller K. et al. (Chiller et al., 2001) |
|  |  | Cohort | Strange P. et al. (Strange et al., 1996) |
|  |  | Book | Arenas Guzman R. (Arenas Guzman, n.d.), Bolognia J. et al. (Bolognia et al., 2015) |
|  | Musculoskeletal System | Review | Godley D. (Godley, 2015), Balato G. et al. (Balato et al., 2021) |
|  |  | Research | Saeed K. et al. (Saeed et al., 2021) |
|  |  | Book | Ochsner P. et al. (Ochsner et al., 2016) |
|  |  | Retrospective | Vander Have K. et al. (Vander Have et al., 2009b) |
| *Streptococcus pyogenes* | Cardiovascular System | Book | Cecchini E. (Cecchini and González Ayala, 2011) |
|  |  | Review | Carapetis J. et al. (Carapetis et al., 2016) |
|  | Integumentary System | Review | Chiller K. et al. (Chiller et al., 2001), Imazio M. et al. (Imazio et al., 2015) |
|  |  | Book | Arenas Guzman R. (Arenas Guzman, n.d.), Bolognia J. et al. (Bolognia et al., 2015) |
|  |  | Case report | Cemeli Cano M. (Cemeli Cano and Beltrán García, 2019) |
| *Coxiella burnetii* | Cardiovascular System | Book | Cecchini E. (Cecchini and González Ayala, 2011) |
|  |  | Review | Eldin C. et al. (Eldin et al., 2017) |
| *Mycoplasma pneumoniae* | Cardiovascular System | Book | Cecchini E. (Cecchini and González Ayala, 2011) |
|  | Respiratory System | Cohort | Huang L. et al. (Huang et al., 2017), Zheng H. et al. (Zheng et al., 2022) |
| *Borrelia burgdorferi* | Cardiovascular System | Book | Cecchini E. (Cecchini and González Ayala, 2011) |
| *Salmonella* | Cardiovascular System | Book | Cecchini E. (Cecchini and González Ayala, 2011) |
|  | Gastrointestinal Tract | Review | Esan O. et al. (Esan et al., 2017) |
| *Corynebacterium diphtheriae* | Cardiovascular System | Book | Cecchini E. (Cecchini and González Ayala, 2011) |
| *Treponema, Borrelia* | Cardiovascular System | Book | Cecchini E. (Cecchini and González Ayala, 2011) |
|  |  | Review | Mac S. et al. (Mac et al., 2020) |
| *Leptospira* | Cardiovascular System | Book | Cecchini E. (Cecchini and González Ayala, 2011) |
|  |  | Review | Albakri A. (Albakri, 2019a) |
| *Pseudomonas aeruginosa* | Respiratory System | Review | Daltro P. et al. (Daltro et al., 2011), Pizzutto S. et al. (Pizzutto et al., 2017) |
| *Helicobacter pylori* | Gastrointestinal Tract | Review | Wang F. et al. (Wang et al., 2014) |
| *Campylobacter jejuni* | Gastrointestinal Tract | Review | Esan O. et al. (Esan et al., 2017) |
| *Yersinia enterocolitica* | Gastrointestinal Tract | Review | Pogreba-Brown K. et al. (Pogreba-Brown et al., 2020) |
| *Shigella spp.* | Gastrointestinal Tract | Review | Pogreba-Brown K. et al. (Pogreba-Brown et al., 2020) |
| *Klebsiella pneumoniae* | Gastrointestinal Tract | Review | Pogreba-Brown K. et al. (Pogreba-Brown et al., 2020) |
| *Enterobacter* spp. | Gastrointestinal Tract | Review | Pogreba-Brown K. et al. (Pogreba-Brown et al., 2020) |
|  | Musculoskeletal System | Review | Godley D. (Godley, 2015), Balato G. et al. (Balato et al., 2021) |
|  |  | Book | Ochsner P. et al. (Ochsner et al., 2016) |
| *Proteus* spp. | Gastrointestinal Tract | Review | Pogreba-Brown K. et al. (Pogreba-Brown et al., 2020) |
| *Group A β-hemolytic Streptococcus* | Musculoskeletal System | Review | Godley D. (Godley, 2015), Balato G. et al. (Balato et al., 2021) |
|  |  | Book | Ochsner P. et al. (Ochsner et al., 2016) |
|  | Renal and Urinary System | Review | Simões e Silva A. et al. (Simões e Silva et al., 2019), San José Gonzáles M. (San José Gonzáles and Méndez Fernández, 2009), Balasubramanian R. (Balasubramanian and Marks, 2017) |
| *Proteus mirabilis* | Renal and Urinary System | Review | Simões e Silva A. et al. (Simões e Silva et al., 2019), San José Gonzáles M. (San José Gonzáles and Méndez Fernández, 2009) |
| *Klebsiella* spp. | Renal and Urinary System | Review | Simões e Silva A. et al. (Simões e Silva et al., 2019), San José Gonzáles M. (San José Gonzáles and Méndez Fernández, 2009) |
| *Staphylococcus saprophyticus* | Renal and Urinary System | Review | Simões e Silva A. et al. (Simões e Silva et al., 2019), San José Gonzáles M. (San José Gonzáles and Méndez Fernández, 2009) |
| *Streptococcus viridians* | Renal and Urinary System | Review | Simões e Silva A. et al. (Simões e Silva et al., 2019), San José Gonzáles M. (San José Gonzáles and Méndez Fernández, 2009), Balasubramanian R. (Balasubramanian and Marks, 2017) |
| *Staphylococcus spp*. | Renal and Urinary System | Review | Simões e Silva A. et al. (Simões e Silva et al., 2019), San José Gonzáles M. (San José Gonzáles and Méndez Fernández, 2009), Balasubramanian R. (Balasubramanian and Marks, 2017) |
| *Pneumococcus* | Renal and Urinary System | Review | Simões e Silva A. et al. (Simões e Silva et al., 2019), San José Gonzáles M. (San José Gonzáles and Méndez Fernández, 2009), Balasubramanian R. (Balasubramanian and Marks, 2017) |
| *Gardnerella vaginalis* | Reproductive System | Review | Ding C. et al. (Ding et al., 2021), Ravel J. et al. (Ravel et al., 2021) |
| *Ureaplasma urealyticum* | Reproductive System | Review | Ding C. et al. (Ding et al., 2021), Ravel J. et al. (Ravel et al., 2021) |
| *Mycoplasma hominis* | Reproductive System | Review | Ding C. et al. (Ding et al., 2021), Ravel J. et al. (Ravel et al., 2021) |
| *Prevotella spp.* | Reproductive System | Review | Ding C. et al. (Ding et al., 2021), Ravel (Ravel et al., 2021) |
| *Herpes types 6* and *7* | Nervous System | Book | Said S. (Said and Kang, 2022) |
| *West Nile* | Nervous System | Book | Said S. (Said and Kang, 2022) |
|  |  | Review | Kumar B. et al. (Kumar et al., 2018) |
| *Varicella-Zoster* | Nervous System | Book | Said S. (Said and Kang, 2022) |
|  | Cardiovascular System | Case report | Alamlih (Alamlih et al., 2021) |
| *Epstein-Barr* | Nervous System | Book | Said S. (Said and Kang, 2022) |
| *Cytomegalovirus* | Nervous System | Book | Said S. (Said and Kang, 2022) |
| *Measles* | Nervous System | Book | Said S. (Said and Kang, 2022) |
| *Mumps* | Nervous System | Book | Said S. (Said and Kang, 2022) |
| *Rubella* | Nervous System | Book | Said S. (Said and Kang, 2022) |
|  |  | Review | Bouthry E. et al. (Bouthry et al., 2014), Lambert N. et al. (Lambert et al., 2015) |
|  |  | Research letter | Cordier A. et al. (Cordier et al., 2012) |
|  | Cardiovascular System | Book | Shukla S. (Shukla and Maraqa, 2022) |
|  | Musculoskeletal System | Review | Banatvala J. (Banatvala and Brown, 2004), Dontigny L. et al. (Dontigny et al., 2018) |
|  |  | Book | Kondamudi N. et al. (Kondamudi and Waymack, 2022) |
| *Eastern equine* | Nervous System | Book | Said S. (Said and Kang, 2022) |
|  |  | Review | Kumar B. et al. (Kumar et al., 2018), Soung A. (Soung and Klein, 2018) |
| W*estern equine*, | Nervous System | Book | Said S. (Said and Kang, 2022) |
|  |  | Review | Kumar B. et al. (Kumar et al., 2018), Soung A. (Soung and Klein, 2018) |
| Dengue | Nervous System | Book | Said S. (Said and Kang, 2022) |
|  | Cardiovascular System | Review | Bäck A. (Bäck and Lundkvist, 2013) |
| *Rabies virus* | Nervous System | Book | Said S. (Said and Kang, 2022) |
|  |  | Review | Kumar B. et al. (Kumar et al., 2018) |
| *Hepatitis B* | Nervous System | Review | Soung A. (Soung and Klein, 2018) |
| *Hepatis C* | Nervous System | Review | Soung A. (Soung and Klein, 2018) |
|  | Cardiovascular System | Research | Faustini A. et al. (Faustini et al., 2010) |
| *SARS* | Nervous System | Review | Gholami M. et al. (Gholami et al., 2021) |
|  | Respiratory System | Review | O’Sullivan O. (O’Sullivan, 2021) |
| *MERS* | Nervous System | Review | Gholami M. et al. (Gholami et al., 2021) |
|  | Respiratory System | Review | O’Sullivan O. (O’Sullivan, 2021) |
| *SARS-CoV-2* | Nervous System | Cross-sectional | Izquierdo-Condoy J.S. et al. (Izquierdo-Condoy et al., 2022a) |
|  |  | Review | Gholami M. et al. (Gholami et al., 2021), Peterson (Peterson et al., 2021) |
|  |  | Case report | Mahmood S. et al. (Mahmood et al., 2022) |
|  |  | Prospective observational | Mattioli F. et al. (Mattioli et al., 2022) |
|  |  | Cohort | Damiano R. et al. (Damiano et al., 2022), Taquet M. et al. (Taquet et al., 2021) |
|  |  | Retrospective longitudinal observational | Romero-Duarte A. et al. (Romero-Duarte et al., 2021) |
|  | Cardiovascular System | Retrospective | Huang L. et al. (Huang et al., 2020) |
|  |  | Cohort | Puntmann V. et al. (Puntmann et al., 2020) |
|  | Respiratory System | Cohort | Zhao Y. et al. (Zhao et al., 2020), Huang C. et al. (Huang et al., 2021) |
|  |  | Longitudinal study | Xiong Q. et al. (Xiong et al., 2021) |
|  |  | Prospective | Pan F. et al. (Pan et al., 2022), Carvalho-Schneider C. et al. (Carvalho-Schneider et al., 2021) |
|  |  | Cross-sectional | Vásconez-González J. et al. (Vásconez-González et al., 2023a), Halpin S. et al. (Halpin et al., 2021) |
|  | Integumentary System | Case sires | Anaya J. et al. (Anaya et al., 2021) |
|  |  | Prospective | Aksoy H. et al. (Aksoy et al., 2021) |
| *HIV* | Nervous System | Review | Brew B. (Brew and Garber, 2018), Hogan C. (Hogan and Wilkins, 2011) |
|  |  | Book | Justiz Vaillant A. et al. (Justiz Vaillant and Gulick, 2022) |
| *Herpes Zoster* | Nervous System | Review | Soung A. (Soung and Klein, 2018) |
|  |  | Prospective observational | Curran D. et al. (Curran et al., 2018) |
| *Chikungunya* | Cardiovascular System | Review | Alvarez M. et al. (Alvarez et al., 2017) |
| *Poliovirus* | Musculoskeletal System | Review | Howard R. (Howard, 2005) |
|  |  | Book | Wolbert J. (Wolbert and Higginbotham, 2022) |
|  |  | Case series | Winter (Winter and Lonstein, 2009) |
| *Taenia solium* | Nervous System | Review | Bustos J. et al. (Bustos et al., 2021), Garcia H. et al. (Garcia et al., 2014), Del Brutto O. (Del Brutto, 2022), Nash T. (Nash and O’Connell, 2020) |
|  |  | Editorial | Sotelo J. (Sotelo, 2003) |
|  | Cardiovascular System | Review | Hidron A. et al. (Hidron et al., 2010) |
|  |  | Cohort | Júnior R. et al. (Júnior et al., 2002) |
| *Toxoplasma gondi* | Nervous System | Book | Kota A. (Kota and Shabbir, 2022), Ayoade F. (Ayoade and Chandranesan, 2022) |
|  |  | Review | Basavaraju A. (Basavaraju, 2016) |
|  |  | Descriptive | Flegr J. et al. (Flegr et al., 2014) |
|  | Cardiovascular System | Review | Hidron A. et al. (Hidron et al., 2010) |
| *Echinococcus* | Nervous System | Prospective | Fares Y. et al. (Fares et al., 2003) |
|  |  | Review | Gessese A. (Gessese, 2020) |
| *Schistosoma* | Nervous System | Review | Carpio (Carpio et al., 2016), Ramos Romero M. (Ramos Romero and Garcia, 2021), Ferrari T. (Ferrari and Moreira, 2011), Carod-Artal F. (Carod-Artal, 2010) |
|  |  | Cross-sectional | Jukes M. et al. (Jukes et al., 2002) |
|  | Cardiovascular System | Review | Hidron A. et al. (Hidron et al., 2010) |
|  | Gastrointestinal Tract | Review | Hidron A. et al. (Hidron et al., 2010) |
| *Plasmodium falciparum* | Nervous System | Review | Carpio A. et al. (Carpio et al., 2016) |
|  |  | Prospective | Oluwayemi (Oluwayemi et al., 2013) |
| *Trypanosoma cruzi* | Cardiovascular System | Review | Meymandi S. et al. (Meymandi et al., 2018), Martín-Escolano J. et al. (Martín-Escolano et al., 2022), Marin-Neto L. et al. (Marin-Neto et al., 2007), Rassi A. (Rassi et al., 2007) |
|  |  | Cohort | Pinto A. et al. (Pinto et al., 2008) |
|  | Gastrointestinal Tract | Review | Matsuda N. et al. (Matsuda et al., 2009), Boyce H. (Boyce and Bakheet, 2005), Bern C. et al. (Bern et al., 2007), Stanaway J. (Stanaway and Roth, 2015) |
|  |  | Cohort | Troncon L. et al. (Troncon et al., 2000) |
| *Filariae* | Cardiovascular System | Review | Hidron A. et al. (Hidron et al., 2010) |
| *Phlebotomus sandflies* | Integumentary System | Review | Bilgic-Temel A. et al. (Bilgic-Temel et al., 2019), Sundar S. (Sundar and Chakravarty, 2015) |
| *Tunga trimamillata* | Integumentary System | Review | Feldmeier H. et al.(Feldmeier et al., 2014) |
|  |  | Cohort | Ariza L. et al. (Ariza et al., 2007) |
|  |  | Letter to the editor | Carretero-Anibarro E. (Carretero-Anibarro and Peñacoba-Masa, 2022) |
|  |  | Case report | Ebrahim A. et al. (Ebrahim et al., 2022) |
| *Diptera fly larvae* | Integumentary System | Review | Calvopina M. (Calvopina et al., 2020), Francesconi F. (Francesconi and Lupi, 2012) |
| *Cryptococcus neoformans* | Nervous System | Descriptive | Lizarazo J. et al. (Lizarazo et al., 2000) |
|  | Cardiovascular System | Review | Albakri A. (Albakri, 2019b) |
| *Candida spp.* | Cardiovascular System | Review | Albakri A. (Albakri, 2019b) |
| *Histoplasma capsulatum* | Cardiovascular System | Review | Kurowski R. (Kurowski and Ostapchuk, 2002) |
| *Histoplasmosis* | Respiratory System | Review | Adenis A. et al. (Adenis et al., 2014) |
| *Trichophyton* | Integumentary System | Prospective | Veien N. et al. (Veien et al., 1994) |
| *Epidermophyton floccosum* | Integumentary System | Prospective | Veien N. et al. (Veien et al., 1994) |
| *Sporothrix schenckii* | Integumentary System | Review | Mahajan V. (Mahajan, 2014) |
| *Scedosporium apiospermum* | Musculoskeletal System | Review | Taj-Aldeen S. et al. (Taj-Aldeen et al., 2015) |
| *Aspergillus* | Musculoskeletal System | Review | Taj-Aldeen S. et al. (Taj-Aldeen et al., 2015) |
| *Candida* | Musculoskeletal System | Review | Taj-Aldeen S. et al. (Taj-Aldeen et al., 2015) |
